# A Novel Four-Gene Prognostic Signature as a Risk Biomarker in Cervical Cancer

**DOI:** 10.1155/2020/4535820

**Published:** 2020-12-14

**Authors:** Jun Wang, Hua Zheng, Yatian Han, Geng Wang, Yanbin Li

**Affiliations:** ^1^Department of Obstetrics and Gynecology, Benxi Central Hospital of China Medical University, Benxi, Liaoning 117022, China; ^2^Key Laboratory of Obstetrics and Gynecology of Higher Education of Liaoning Province, Shenyang, Liaoning 110000, China; ^3^Key Laboratory of Maternal-Fetal Medicine of Liaoning Province, Shenyang, Liaoning 110000, China; ^4^Department of Oncology, The Affiliated Benxi Jinshan Hospital of Dalian Medical University, Benxi, Liaoning 117022, China; ^5^Department of Cardiology, Benxi Central Hospital of China Medical University, Benxi, Liaoning 117022, China

## Abstract

**Background:**

Cervical cancer (CC) is a major malignancy affecting women worldwide, with limited treatment options for patients with advanced disease. The aim of this study was to identify novel prognostic biomarkers for CC.

**Methods:**

RNA-Seq data from four Gene Expression Omnibus datasets (GSE5787, GSE6791, GSE26511, and GSE63514) were used to identify differentially expressed genes (DEGs) between CC and normal cervical tissues. Functional and enrichment analyses of the DEGs were performed using the Search Tool for the Retrieval of Interacting Genes/Proteins (STRING) database and the Database for Annotation, Visualization, and Integrated Discovery (DAVID). The Oncomine database, Cytoscape software, and Kaplan-Meier survival analyses were used for in-depth screening of hub DEGs. The Cox regression was then used to develop a prognostic signature, which was in turn used to create a nomogram.

**Results:**

A total of 207 DEGs were identified in the tissue samples, eight of which were prognostically significant in terms of overall survival (OS). Thereafter, a novel four-gene signature consisting of DSG2, MMP1, SPP1, and MCM2 was developed and validated using stepwise Cox analysis. The area under the receiver operating characteristic (ROC) curve (AUC) values were 0.785, 0.609, and 0.686 in the training, verification, and combination groups, respectively. The protein expression levels of the four genes were well validated by the western blotting. Moreover, the nomogram analysis showed that a combination of this four-gene signature plus lymph node metastasis (LNM) status effectively predicted the 1- and 3-year OS probabilities of CC patients with accuracies of 69.01% and 83.93%, respectively.

**Conclusions:**

We developed a four-gene signature that can accurately predict the prognosis in terms of OS, of CC patients, and could be a valuable tool for designing treatment strategies.

## 1. Introduction

Cervical cancer (CC) is one of the most common malignancies and a major cause of cancer-related death among women globally [1]. Recently, the incidence of CC has gradually increased, particularly among younger women (35-39 years old) [2]. In >95% of cases, CC is closely related to the presence of persistent high-risk types of human papillomavirus (HPV) [3]. Although the HPV vaccine is effective for the prevention of CC, it does not cover all pathogens associated with CC, and it is not universally available to women, especially those in low- and middle-income countries where there is high incidence of and mortality due to a lack of effective screening and treatment of CC [4, 5]. Therefore, informative biomarkers are needed for CC diagnosis and prognosis prediction.

High-throughput sequencing is an effective method that can be used to screen biomarkers for cancers. With advances in microarray technology, small changes at the level of transcription in addition to dysregulation of posttranscriptional signaling in CC can be detected [6]. For instance, Yan et al. used cDNA microarray analysis to show that CXCL8 is overexpressed in cervical cancer tissues relative to tissues from cervical intraepithelial lesions [7]. And Zheng found that minichromosome maintenance protein 2 (MCM2) could significantly improve the sensitivity and specificity of the diagnosis of cervical lesions linked to HPV infection [8]. However, few studies have identified prognostic and predictive signatures by combining with multigenes, and thus a comprehensive analysis for the identification of a robust signature for CC is still needed.

To explore potential biomarkers of poor overall survival (OS) among CC patients in greater detail, we used four Gene Expression Omnibus (GEO) datasets (GSE5787, GSE6791, GSE26511 and GSE63514) to improve the accuracy of the results. By screening and conducting validation based on the Database for Annotation, Visualization, and Integrated Discovery (DAVID) database, Oncomine database, Search Tool for the Retrieval of Interacting Genes/Proteins (STRING) database, and Molecular Complex Detection (MCODE) plug-in in Cytoscape, 40 hub differentially expressed genes (DEGs) were identified between CC and normal cervical tissues. Thereafter, the mRNA expression data on the hub DEGs in CC patients (who had corresponding clinical data) in The Cancer Genome Atlas- (TCGA-) CC cohort were used together with a stepwise Cox regression analysis to develop a robust four-gene prognostic signature. This signature involved desmoglein 2 (DSG2), matrix metallopeptidase 1 (MMP1), secreted phosphoprotein 1 (SPP1), and MCM2. Nomogram analysis suggested that this four-gene signature and lymph node metastasis (LNM) status could accurately predict 1- and 3-year overall survival (OS) among CC patients. In summary, the novel four-gene signature is not only about CC pathogenesis but also represents a new method for prognostic evaluation of this type of cancer.

## 2. Materials and Methods

### 2.1. Study Design

We collected and collated messenger RNA (mRNA) expression datasets based on the Affymetrix Human Genome U133 Plus 2.0 Array platform in the GEO database (https://www.ncbi.nlm.nih.gov/gds/). Probe information for the microarrays was read and normalized using the “affy” package in *R* software. The batch effects in the microarray experiments were removed using the “sva” package [9]. Principal component analysis (PCA) was used to assess whether the samples in each group (CC tissues [*n* = 98] and normal cervical tissues [*n* = 32]) were clustered prior to using the samples to identify DEGs. Thereafter, both mRNA expression data and corresponding clinical data for patients with CC (*n* = 304) were obtained from the TCGA database (https://cancergenome.nih.gov) for additional analyses.

Cervical samples were collected from 66 surgical patients hospitalized at the Department of Gynecology of Benxi Central Hospital from March 2019 to July 2020. The 66 samples included those from 34 malignant tumors originating from cervical squamous cell carcinoma, 2 cervical adenocarcinoma, and 30 normal cervical tissues. The median ages of patients who supplied malignant and normal cervical samples were 53.3 (41-72) and 49.5 (30-78) years, respectively. Patients were not treated with radiotherapy, chemotherapy, or hormone therapy. All patients were informed about the experiments and signed informed consent forms. The tissue-associated experiments were approved by the Clinical Research Ethics Committee of Benxi Central Hospital of China Medical University.

### 2.2. Identification of DEGs

The differential expression matrix of the GEO samples included in the analysis was extracted from the total gene expression matrix, with DEGs between the CC and normal cervical tissues identified using the “limma” package. Genes with ∣log2(fold change) | >1.5 and *p* < 0.05 were considered to be potentially relevant DEGs and were subjected to further analysis.

### 2.3. Functional and Pathway Enrichment Analyses

To analyse the DEGs in terms of functional and pathway enrichment, Gene Ontology (GO) and Kyoto Encyclopedia of Genes and Genomes (KEGG) analyses were performed using the DAVID database (https://david.ncifcrf.gov). The results were visualized using the “GOplot” package, and the terms were sorted by *p* value.

### 2.4. Protein-Protein Interaction (PPI) Network Construction

The STRING database (https://string-db.org/) is an online search tool that is frequently used to identify regulatory hub genes. Cytoscape (version 3.6.1) allows visualization and analysis of PPI networks based on the STRING database. We identified candidate hub DEGs using the Cytoscape plug-in MCODE with degree cutoff = 2, node density cutoff = 0.1, node score cutoff = 0.2, and k − core = 2.

### 2.5. Expression Validation and Survival Analysis of the Individual Hub DEGs

To validate the candidate hub DEGs, the mRNA expression of these DEGs was validated using the Oncomine database (https://www.oncomine.org/), employing a threshold *p* value of 1 × 10 − 4 and a fold change of 2 in five Oncomine microarray datasets. Additionally, the TCGA samples (*n* = 304) were divided into high- and low-expression groups using the median expression level of each individual candidate hub DEG as the cutoff value, and Kaplan-Meier survival analysis was then performed for the high- and low-expression groups using the “survival” package in *R* software.

Total protein was extracted using RIPA lysis buffer containing the protease inhibitor phenylmethylsulfonyl fluoride (PMSF) and a phosphatase inhibitor (P0013B; Beyotime, China). Protein concentrations were measured using a bicinchoninic acid protein assay kit (Pierce Biotechnology, Waltham, MA, USA). Equal amounts of proteins were separated by 6-12% sodium dodecyl sulfate-polyacrylamide gel electrophoresis and transferred onto a polyvinylidene difluoride membrane. The membrane was blocked with 5% bovine serum albumin and probed with primary antibodies at 4°C overnight (Table SI). After washing the membrane with Tris-buffered saline containing Tween-20, it was incubated with horseradish peroxidase-conjugated secondary antibodies (anti-rabbit IgG, 1 : 5,000, CST, USA) for 2 h at room temperature. The antigen-antibody complexes were visualized using enhanced chemiluminescence plus reagent (Millipore, Billerica, MA, USA). A Gel-Pro Analyser (Bio-Rad) was used for band densitometry, using *β*-actin as a reference.

### 2.6. Cox Proportional Hazards Regression Model and Risk Score

The TCGA-CC samples (*n* = 304) were randomly divided into a training group (*n* = 152) and a verification group (*n* = 152). We then carried out a stepwise Cox regression analysis to identify significant hub DEGs and establish the best model based on the Akaike information criterion (AIC), with the verification and combination groups used for validation. Based on the best model, we then calculated the risk scores for predicting poor OS among CC patients using the following formula (which was used to create the four-gene prognostic signature):

(1) $$\begin{array}{c} \text{Risk score}=\overset{\infty }{\underset{n=1}{\sum }}\left(e_{n}*\beta_{n}\right), \end{array}$$

where *β* is the estimated stepwise Cox regression coefficient of the mRNA, and *e* is the mRNA expression level.

Based on the median risk score, CC patients were categorized into high- and low-score (risk) groups. A receiver operating characteristic (ROC) curve analysis was used to assess the prognostic performance of the four-gene prognostic signature. Kaplan-Meier survival curves and the log-rank test were used to determine associations between the risk score and OS among patients with CC.

### 2.7. Independence of Final Signature from Conventional Clinical Feature

Using CC patients (*n* = 304) in the TCGA-CC cohort with survival status information and detailed clinicopathological information, comprising age, LNM status, Fédération Internationale de Gynécologie et d'Obstétrique (FIGO) stage, and tumor grade, univariate and multivariate Cox regression analyses were conducted to identify whether the four-gene prognostic signature was independent of conventional clinical characteristics.

### 2.8. Analysis of the Nomogram for Predicting OS

On the basis of the independent prognostic factors identified in the final multivariate Cox regression analysis, nomograms were used to predict OS (*n* = 142, 56, and 29 for the 1-, 3-, and 5-year analyses, respectively) among CC patients in the TCGA-CC cohort. The nomograms were visually assessed using calibration plots comparing the predicted and actual survival probabilities among CC patients. The prognostic performance of the nomogram was determined based on the area under the ROC curve (AUC), which can range from 0.5 (no discrimination) to 1 (perfect discrimination).

### 2.9. Statistical Analysis

*R* software (version 3.5.3) with *R* Studio (version 1.1.463) and the Perl scripting tool (version 5.26.3) were used for data analysis. Data used for comparison between two groups are presented as the mean ± standard error of the mean (SEM). All data were checked for normal distribution and homogeneity of variance using the Shapiro-Wilk test and the Levene test, respectively. Two-tailed Student's unpaired *t*-test was used to compare means between two groups. A violin plot was used to show the differential distribution of the risk scores in each of the subgroups stratified by clinical features (age, tumour grade, FIGO stage, and LNM status). The optimal cutoff age of TCGA-CC patients was determined based on the survival status using X-tile software (version 3.6.1) [10]. Kaplan-Meier survival analysis was used to assess the association between median risk score and survival (including in the two age subgroups), with the results presented using survival curves and significant differences being determined using the log-rank test. Significance was defined as *p* < 0.05.

## 3. Results

### 3.1. Screening for DEGs

**Figure 1:**
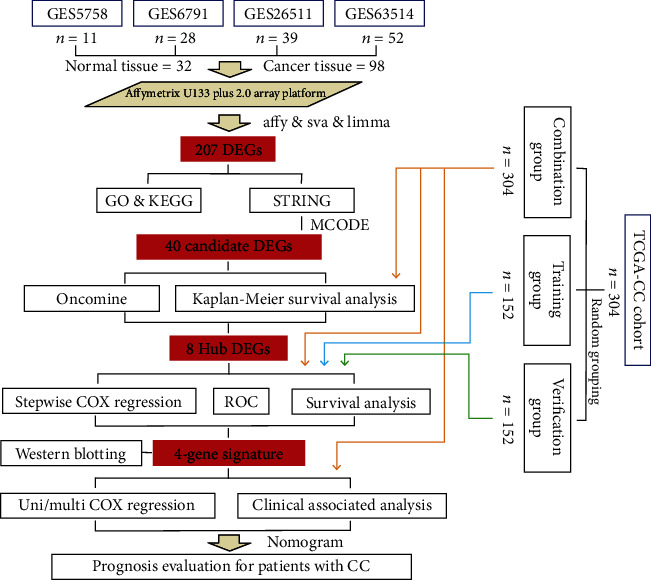
Flow chart of this study. CC: cervical cancer; DEG: differentially expressed gene; ROC: receiver operating characteristic.

**Figure 2:**
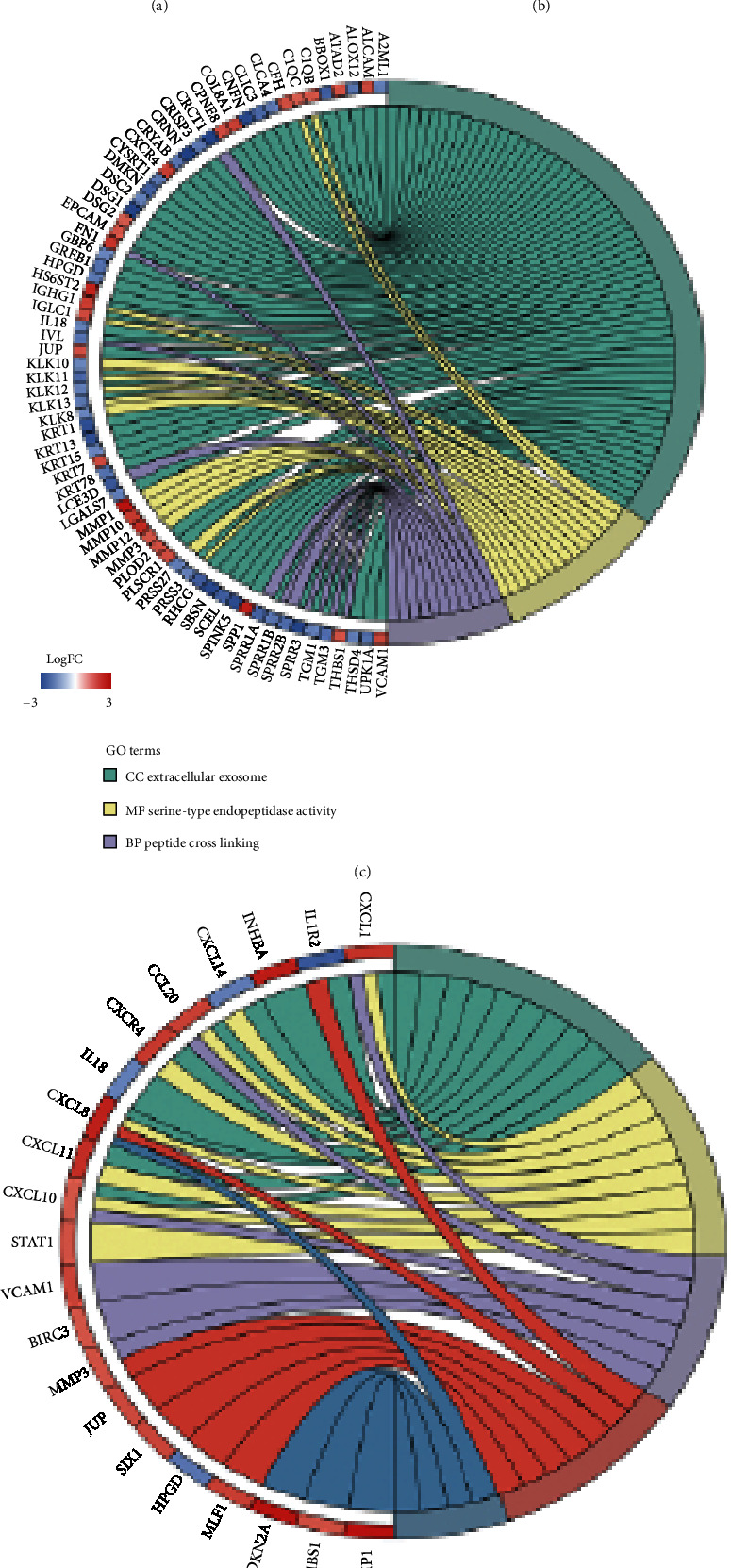
Screening for DEGs in samples from patients with CC. (a) PCA plot showing significant differential clustering between CC (red spheres) and normal cervical (yellow spheres) tissue samples. (b) Volcano plots depicting changes in mRNA expression between the normal cervical tissue (*n* = 32) and CC (*n* = 98) groups. There were 106 and 101 mRNAs with ∣log2(fold change) | >1.5 that were significantly (*p* < 0.05) upregulated (red) and downregulated (blue) in the CC group compared to the normal cervical tissue group. (c, d) GO and KEGG enrichment analyses of DEGs in CC samples. CC: cervical cancer; DEG: differentially expressed gene; PCA: principal component analysis; FC: fold change; GO: Gene Ontology; KEGG: Kyoto Encyclopedia of Genes and Genomes.

The study was constructed as shown in the flow chart in Figure 1. To identify prognostic genes that play a role in CC pathogenesis, we used CC tissues and normal cervical tissues (Table SII). We stabilized the error rate estimates and improved the reproducibility of the gene expression matrix using surrogate variables for removing batch effects (Figure S1). PCA showed that the two groups of samples were obviously clustered (Figure 2(a)). A total of 207 DEGs between the two groups were observed, with 106 being upregulated and 101 being downregulated (Figure 2(b), Table SIII).

### 3.2. Functional and Pathway Enrichment Analyses of DEGs

GO and KEGG analyses were performed using the DAVID database. Regarding GO terms, the DEGs were primarily enriched in extracellular exosome (58 proteins), serine-type endopeptidase activity (16 proteins), and peptide crosslinking (11 proteins) (Figure 2(c)). KEGG analysis revealed that the DEGs were enriched in several pathways, such as cytokine-cytokine receptor interaction (10 proteins), chemokine signaling (8 proteins), and tumor necrosis factor (TNF) signaling (6 proteins), as well as transcriptional dysregulation in various cancers, such as bladder cancer (4 proteins) (Figure 2(d)).

### 3.3. Identification of Hub DEGs in CC

**Figure 3:**
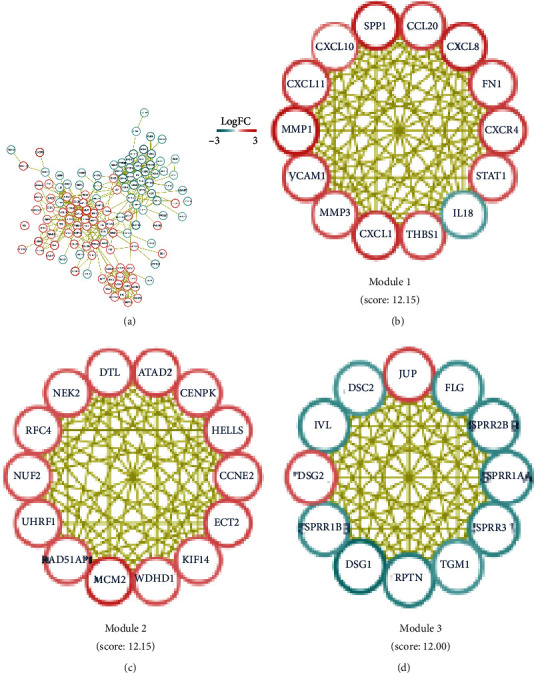
PPI network of DEGs in CC. (a) PPI network demonstrating the interactions between upregulated (red) and downregulated (green) proteins. (b)–(d) The three most significant submodules (MCODE scores ≥ 10) contained 14 (b), 14 (c), and 12 (d) candidate DEGs, respectively. Node color is positively related to fold change in expression; yellow link indicates specific or meaningful association among nodes. CC: cervical cancer; DEG: differentially expressed gene; PPI: protein-protein interaction.

**Figure 4:**
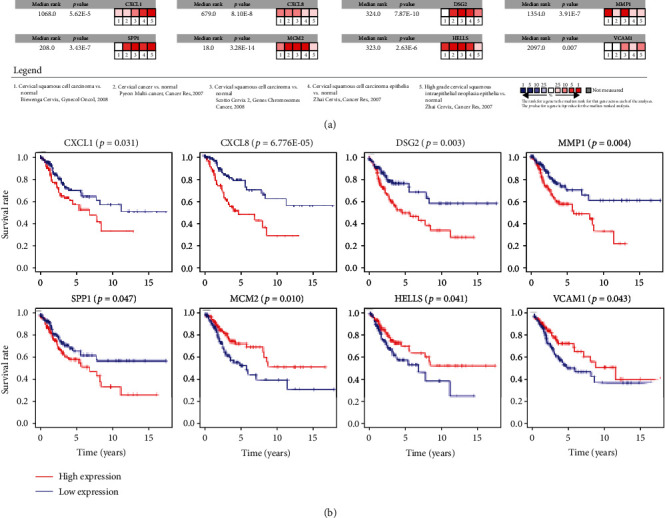
In-depth screening for hub DEGs in CC. (a) mRNA expression levels of candidate hub DEGs evaluated using the Oncomine microarray database. (b) Kaplan-Meier survival analysis and the log-rank test. The mRNA levels of CXCL1 (*p* = 0.031), CXCL8 (*p* < 0.001), DSG2 (*p* = 0.003), MMP1 (*p* = 0.004), SPP1 (*p* = 0.047), MCM2 (*p* = 0.010), HELLS (*p* = 0.041), and VCAM1 (*p* = 0.043) were significantly associated with OS among CC patients. CC: cervical cancer; DEG: differentially expressed gene; OS: overall survival.

Although enrichment analyses reveal the biological processes and pathways related to DEGs, they do not provide information about interactions among the DEGs. Thus, we examined the interactions among the proteins using STRING and visualized the PPI network using Cytoscape software. To construct the PPI network, 112 significantly enriched DEGs were submitted to STRING (Figure 3(a)), and the PPI network was subsequently imported into Cytoscape to construct the subnetworks. Using the MCODE plug-in in Cytoscape, we analyzed the top three submodules (MCODE scores ≥ 10) of proteins to identify the hub DEGs. There were 40 hub DEGs in these modules, with those in modules 1 and 2 primarily being upregulated DEGs and those in module 3 primarily being downregulated DEGs (Figures 3(b)–3(d)). The Oncomine coexpression analysis showed that the mRNA expression levels of 22 of the candidate hub DEGs were consistent with our initial analyses (Figure 4(a) and Figure S2).

To examine the hub DEGs in greater detail, TCGA CC samples (*n* = 304) were used for survival analyses of the individual hub DEGs (with a cutoff value of *p* < 0.05). The expression of eight hub DEGs (CXCL1 (*p* = 0.031), CXCL8 (*p* = 6.776E − 05), DSG2 (*p* = 0.003), MMP1 (*p* = 0.004), SPP1 (*p* = 0.047), MCM2 (*p* = 0.010), lymphoid-specific helicase [HELLS] (*p* = 0.041), and vascular cell adhesion molecule 1 [VCAM1] (*p* = 0.043)) was significantly associated with OS among CC patients. Interestingly, MCM2, HELLS, and VCAM1 upregulation played protective roles (Figure 4(b) and Figure S3).

### 3.4. Cox Proportional Hazards Model and Risk Score

The TCGA-CC samples (*n* = 304) were randomly divided into a training group (*n* = 152) and a verification group (*n* = 152). Among the 304 patients, 223 (73.4%) and 87 (28.3%) had complete follow-up data of clinical features for at least 1- and 3-years, respectively, but only 40 (13.2%) had detailed follow-up data for ≥5 years (Table SIV). There were no significant differences in age, race, tumor grade, FIGO stage, or LNM status between the training and verification groups (Table 1). Next, we assessed the significance of the eight abovementioned hub DEGs in a Cox proportional hazards model and consequently developed a novel four-gene prognostic signature. This signature allowed us to determine the high- and low-risk patients, as follows:

Risk score = (0.58∗expression value of DSG2) + (0.27∗expression value of MMP1) + (0.33∗expression value of SPP1) + (−0.48∗expression value of MCM2)

**Figure 5:**
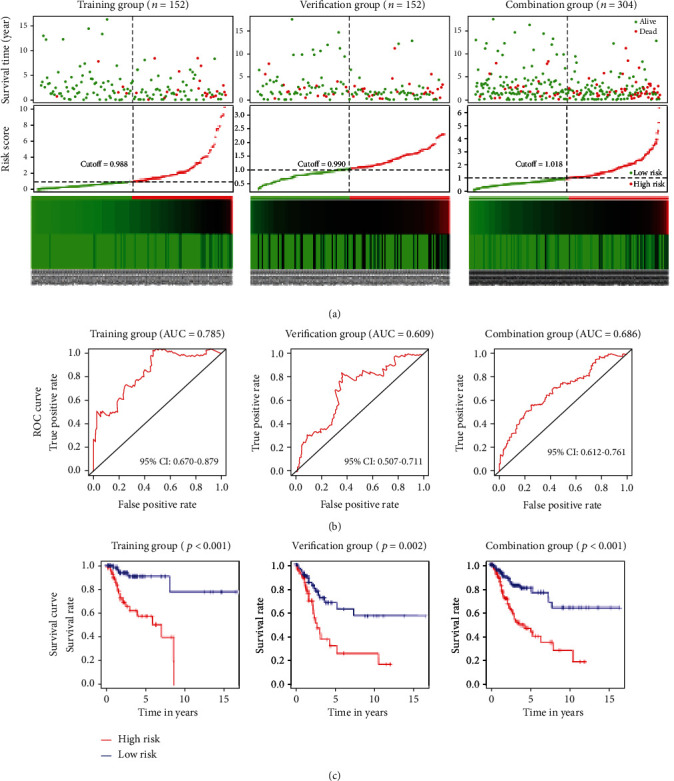
Construction of a four-gene prognostic signature for CC. (a) Survival status of patients with CC and four-gene signature risk score distribution for the training, verification, and combination groups. The black-dotted line represents the median cutoff. (b) AUC of the ROC curve evaluating the ability of the four-gene signature to predict prognosis in terms of OS. (c) Kaplan-Meier curves of the four-gene signature indicating that high-risk patients had shorter survival than low-risk patients. AUC: area under the curve; CC: cervical cancer; CI: confidence interval; OS: overall survival; ROC: receiver operating characteristic.

With validation using the verification and combination groups, we built the best fitting Cox proportional hazards model using a combination of four high-power prognostic genes (DSG2, MMP1, SPP1, and MCM2) (Figure 5(a)). The ROC curves showed that this four-gene signature achieved AUC values of 0.785 (95% confidence interval [CI]: 0.670-0.879), 0.609 (95% CI: 0.507-0.711), and 0.686 (95% CI: 0.612-0.761) for the training, verification, and combination groups, respectively (Figure 5(b)). These outcomes suggest that this four-gene signature demonstrates good performance regarding the prediction of OS among CC patients (Figure 5(c)).

**Figure 6:**
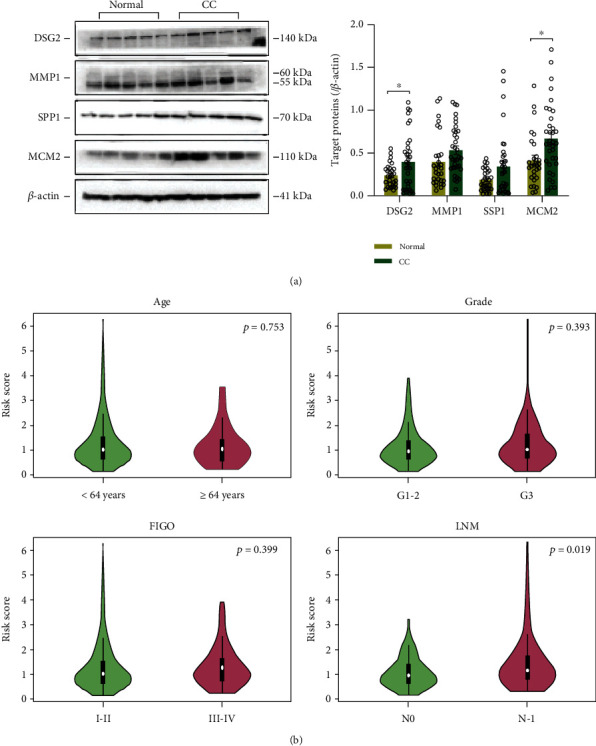
Western blotting and clinical analyses. (a) Immunoblots for four-gene prognostic signature associated proteins DSG2 (CC *n* = 34, normal *n* = 29), MMP1 (CC *n* = 35, normal *n* = 29), SPP1 (CC *n* = 36, normal *n* = 30), MCM2 (CC *n* = 35, normal *n* = 29), and quantitative data. ^∗^*p* < 0.05 vs. normal. (b) Violin plots showing differences in the mean risk score in CC patients stratified by age (mean ± SEM: 1.225 ± 0.054 vs 1.183 ± 0.111), tumor grade (1.155 ± 0.062 vs 1.240 ± 0.081), FIGO stage (1.194 ± 0.057 vs 1.295 ± 0.098), and LNM status (1.056 ± 0.053 vs 1.341 ± 0.138) (< 64 years old [*n* = 256], ≥ 64 years old [*n* = 48]; grades G1-G2 [*n* = 153], G3 [*n* = 119]; stages I-II [*n* = 189], III-IV [*n* = 108]; LNM N0 [*n* = 133], LNM [N1 *n* = 60]). *p* < 0.05 represents significance between corresponding subgroups, according to unpaired Student's *t-*test. CC: cervical cancer; FIGO: Fédération Internationale de Gynécologie et d'Obstétrique; LNM: lymph node metastasis (N0 = negative, N1 = positive).

Furthermore, the protein levels of the four genes were confirmed by western blot analysis. The protein expression levels of DSG2 (0.238 ± 0.025 vs 0.396 ± 0.056, *p* = 0.018) and MCM2 (0.413 ± 0.081 vs 0.667 ± 0.077, *p* = 0.007) were significantly higher in CC than in normal cervical tissues, and the protein expression trend of MMP1 (0.397 ± 0.058 vs 0.534 ± 0.048, *p* = 0.071) and SPP1 (0.328 ± 0.116 vs 0.340 ± 0.061, *p* = 0.925) also seemed increased, consistent with the abovementioned mRNA data (Figure 6(a)).

### 3.5. OS Prediction and Evaluation

To further evaluate whether the four-gene prognostic signature can serve as a prognostic factor, we performed univariate and multivariate Cox regression analyses comparing high- and low-risk CC patients. Covariates besides the risk score included clinical risk factors such as age, tumor grade, FIGO stage, and LNM status (Figure S4). The univariate Cox regression analysis showed that the risk score (hazard ratio [HR]: 3.186; 95% CI: 1.513-6.711; *p* = 0.003) and LNM status (HR: 2.886; 95% CI: 1.435-5.803; *p* = 0.003) were risk factors, while the multivariate Cox regression analysis confirmed that both risk score (HR: 2.743; 95% CI: 1.285-5.856; *p* = 0.009) and LNM status (HR: 2.660; 95% CI: 1.290-5.489; *p* = 0.008) were independent risk factors (Table 2).

The risk score was then compared between the pairs of subgroups stratified by clinical features to explore whether it was significantly different between the various subgroups. It was only significantly different between LNM-negative and LNM-positive patients, being higher in the latter (1.056 ± 0.053 vs 1.341 ± 0.138, *p* = 0.019) (Figure 6(b)).

**Figure 7:**
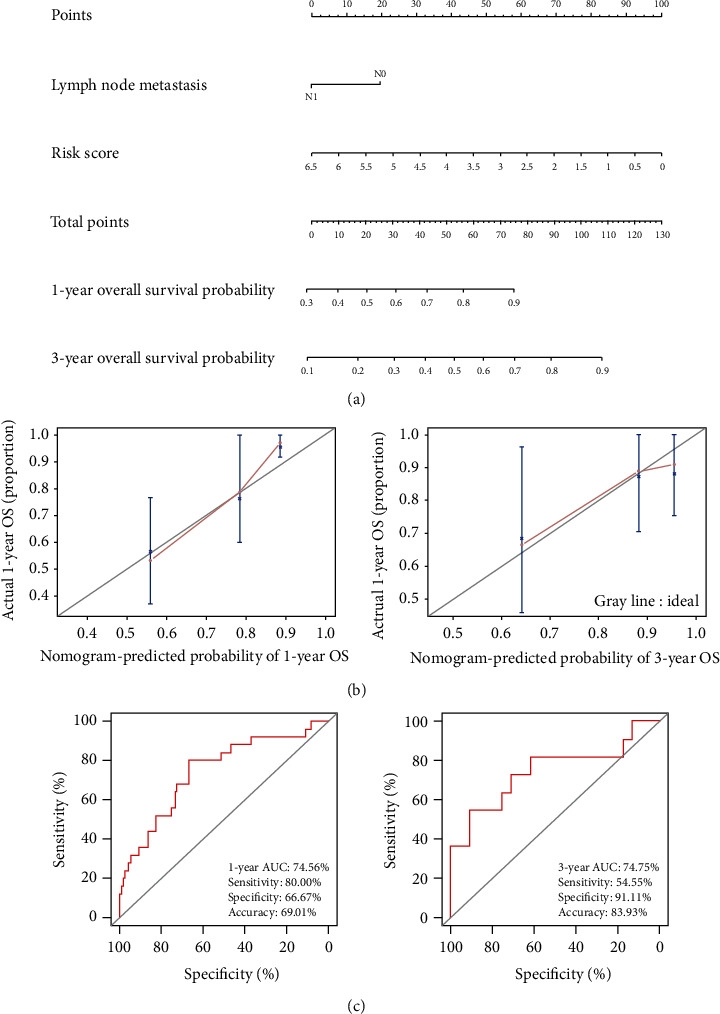
Analysis of the nomogram for predicting OS among patients with CC. (a) Nomogram of two independent risk factors (four-gene signature and LNM status); the scores for each variable were added to obtain the total score for predicting the 1- and 3-year OS of patients with CC. (b) Calibration plots for the nomogram predicting 1-year OS (*n* = 142) and 3-year OS (*n* = 56). The reference line represents a perfect match between the predicted and actual survival probabilities. (c) AUC of the ROC curve verifying the prognostic accuracy of the nomogram for predicting 1- and 3-year OS. AUC: area under the curve; CC: cervical cancer; LNM: lymph node metastasis; OS: overall survival; ROC: receiver operating characteristic.

We also constructed a nomogram to predict 1- and 3-year OS for CC patients using the four-gene signature and LNM status (Figure 7(a)). In the nomogram model, although the four-gene signature could independently increase the agreement between the predicted and actual probabilities without LNM status for the 1- and 3-year OS analyses of CC based on the TCGA-CC cohort, the AUCs were only approximately 0.701 (95% CI: 0.579-0.823, *n* = 142) and 0.610 (95% CI: 0.420-0.800, *n* = 56), respectively (Figure S5). By contrast, the combination of the four-gene signature plus LNM status showed better agreement between the predicted and actual probabilities regarding 1- and 3-year but not 5-year OS (Figure 7(b)). The AUC values for 1- and 3-year OS were 0.746 (95% CI: 0.635-0.857) and 0.748 (95% CI: 0.551-0.944), respectively, and the prognostic accuracy values were 69.01% and 83.93%, respectively (Figure 7(c)).

## 4. Discussion

CC is a malignant disease and is the fourth most frequent cancer in the world, with 569,847 new cases and 311,365 deaths in 2018 [11]. When detected early, CC is highly treatable, and these patients have high survival rates and good quality of life. During tumorigenesis as well as during cancer development, mRNA expression levels can exhibit minor changes. During CC progression, multiple mRNAs have been shown to be dysregulated [12, 13], although the prognostic value of multi-mRNA signatures based on samples from CC patients remains unclear. In the present study, we developed a novel four-gene signature and validated it as a biomarker for early diagnosis and prediction of 1- and 3-year OS among CC patients. This four-gene signature might constitute an important step forward for treatment decisions and may predict more accurate and individualized prognoses for CC patients. This four-gene signature also provides a basis for future experimental research.

Several studies have examined the potential of multi-mRNA signatures for clinical research on CC. Huang et al. provided a prediction model of CC recurrence based on the expression patterns of seven genes (ubiquitin-like 3 [UBL3], fibroblast growth factor 3 [FGF3], BMI1 polycomb ring finger [BMI1], platelet-derived growth factor receptor [PDGFRA], protein tyrosine phosphatase, receptor type, F [PTPRF], replication factor C [RFC4], and nucleolar protein 7 [NOL7]) [14], while Ding et al. developed a prediction model of CC survival based on the expression of three genes (methionine sulfoxide reductase B3 [MSRB3], centromere protein M [CENPM], and Zic family member 2 [ZIC2]) [15]. Although all studies developed a prediction tool for CC prognosis based on completely different genes, our research paid more attention to the integration of the four-gene prognostic signature and lymph node status. The recent change to FIGO staging of CC cases reflects the importance of LNM status, and several reports have demonstrated that positive pathologic LNM is more strongly associated with the survival rate than other risk factors such as age, histology, and clinical stage [16, 17], although LNM status alone may not predict CC prognosis. In the present study, we demonstrated that both the four-gene signature and LNM status had prognostic value for CC, and we developed a nomogram that integrated the four-gene prognostic signature and LNM status to accurately predict the 1- and 3-year OS rates of patients with CC. According to the four-gene prognostic signature, DSG2, MMP1, and SPP1 are risk factors, whereas MCM2 is a protective factor for patients with CC.

DSG2 is a member of the desmoglein family and the cadherin cell adhesion molecule superfamily. Although its precise role in CC is unclear, it is thought to be involved in the development of several types of cancers [18, 19]. It is related to keratinization, developmental biology, and mitogen-activated protein kinase (MAPK) signaling pathways. Our analysis revealed that patients with high DSG2 expression had poorer prognosis than those with low expression, suggesting that it may play a role in predicting prognosis in terms of poor OS among CC patients.

MMP1, also known as interstitial collagenase, is located on chromosome 11q22.3 and belongs to the matrix metalloproteinase family. It can promote tumor invasion and metastasis through mechanisms involving angiogenesis and immune evasion [20]. The overexpression of MMP1 is strongly associated with unfavorable prognosis in multiple malignancies including breast cancer, oesophageal squamous cell carcinoma, and ovarian cancer [21–23]. MMP1 has also previously been proposed as a risk factor in CC [24, 25].

SPP1 participates in the regulation of tumor-associated angiogenesis and inflammation [26]. Previous bioinformatic analysis showed that SPP1 is closely related to the incidence and poor prognosis of CC [27], which is consistent with our findings. Moreover, SPP1 downregulation improves the cisplatin sensitivity of HeLa cells by inhibiting the activity of the phosphoinositide 3-kinase (PI3K)/Akt signaling pathway [28].

MCM2 is a component of the DNA replication licensing complex (MCM2-7) that has been found to mainly localize to the nucleus in eukaryotic cells [29]. The overexpression of MCM2 frequently occurs in CC, particularly in cases involving persistent infection with high-risk HPV [8]. MCM2 has been reported to promote tumor proliferation by mediating DNA replication, initiation, and elongation [30]. In contrast, in our study, MCM2 played a protective role in CC progression; although consistent with previous studies [30, 31], we also observed high expression levels of MCM2 in CC tissues. Aihemaiti et al. reported that cytoplasmic rather than nuclear accumulation of MCM2 is related to improved survival for patients with ovarian clear cell carcinoma [32], which may be associated with MCM2-mediated DNA damage-induced apoptosis [33, 34]. This pathway may also function in CC, although additional investigation is needed to explore this possibility.

There are some limitations to this study: (i) the sample size was small. (ii) The patients were largely European and American, and few Asian patients were included in the GEO datasets and the TCGA-CC cohort; however, we are currently collecting tissues from patients treated at the Obstetrics and Gynecology Department of Benxi Central Hospital in China for further analysis. (iii) Additional investigation based on different histological types is needed both to define the detailed mechanisms of the hub DEGs (particularly DSG2 and MCM2) in CC pathogenesis and to validate the relationship of the four-gene signature with CC prognosis in a larger cohort.

## 5. Conclusion

In summary, the mRNA expression levels of four hub DEGs (DSG2, MMP1, SPP1, and MCM2) were significantly associated with OS among CC patients, and the novel four-gene signature could have substantial prognostic value, allowing prediction of OS among patients with CC. The efficacy of the four-gene signature for patients with CC is promising and warrants additional investigation.

## Figures and Tables

**Table 1:** Clinical features of CC patients in the training and verification groups.

Feature	Training group (*n* = 152)	Verification group (*n* = 152)	*p* value
Age			
≤64	129	136	0.304
>64	23	16	
Grade			
G1-G2	79	74	0.834
G3	58	61	
Unknown	15	17	
FIGO stage			
I-II	112	119	0.607
III-IV	36	29	
Unknown	4	4	
LNM status			
Positive	60	73	0.278
Negative	34	26	
Unknown	58	53	
Race			
White	118	121	0.700
Asian	9	11	
Other	4	5	
Not reported	21	15	

CC: cervical cancer; FIGO: Fédération Internationale de Gynécologie et d'Obstétrique; LNM: lymph node metastasis.

**Table 2:** Univariate/multivariate Cox regression analyses.

Variables	Univariate analysis			Multivariate analysis		
	HR	95% CI	*p* value	HR	95% CI	*p* value
Age						
≤ 64/>64	2.567	0.888-7.419	0.082	1.543	0.514-4.631	0.439
Grade						
G1-2/G3	1.022	0.502-2.077	0.953	0.979	0.479-2.002	0.953
FIGO stage						
I-II/III-IV	0.948	0.288-3.124	0.931	0.764	0.219-2.669	0.673
LNM status						
Positive/negative	2.886	1.435-5.803	0.003	2.660	1.290-5.489	0.008
Risk score						
High/low	3.186	1.513-6.711	0.003	2.743	1.285-5.856	0.009

CI: confidence interval; FIGO: Fédération Internationale de Gynécologie et d'Obstétrique; HR: hazard ratio; LNM: lymph node metastasis.

## Data Availability

Publicly available datasets were analysed in this study, which can be found here: (1) https://www.ncbi.nlm.nih.gov/gds/?term=2. https://portal.gdc.cancer.gov/.

## Supplementary Materials

Supplementary Materials Table SI: antibodies for western blotting. Table SII: clinical features of CC patients from GEO datasets. Table SIII: differentially expressed genes. Table SIV: clinical features of CC patients from the TCGA-CC cohort.
