# Supplementary material for: A Novel Four-Gene Prognostic Signature as a Risk Biomarker in Cervical Cancer
Source: Int J Genomics. 2020 Dec 14;2020:4535820. doi: 10.1155/2020/4535820 (PMC7758149; doi:10.1155/2020/4535820)
Supplement: Supplementary Materials — Table SI: antibodies for western blotting. Table SII: clinical features of CC patients from GEO datasets. Table SIII: differentially expressed genes. Table SIV: clinical features of CC patients from the TCGA-CC cohort. [file 4535820.f1.docx]

**Supplementary Figures**

**
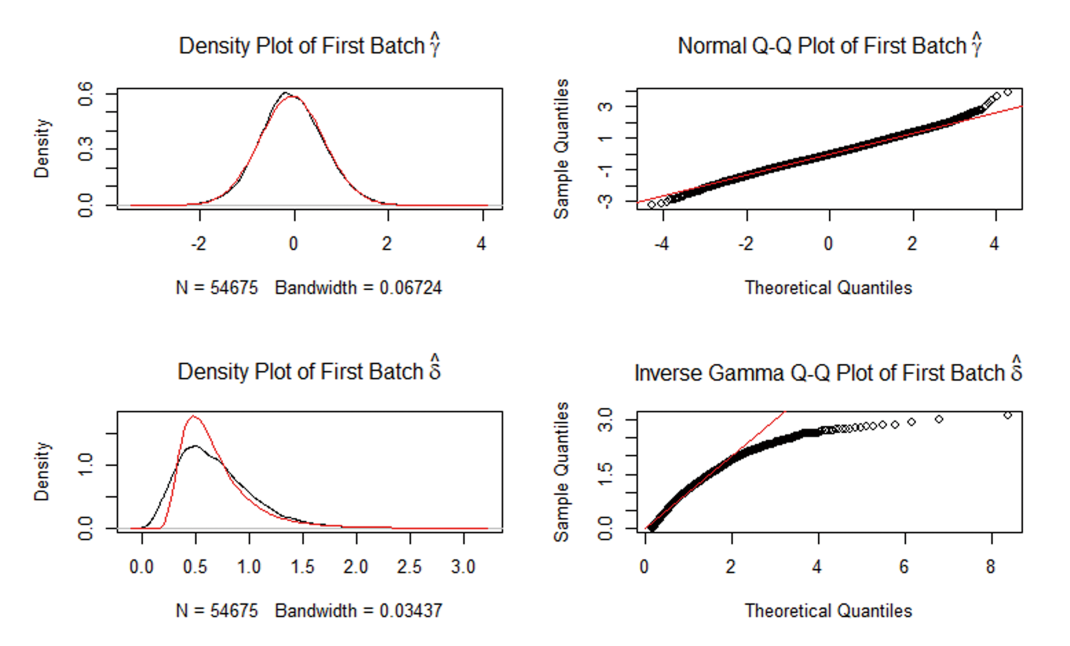
**

**FIGURE S1**


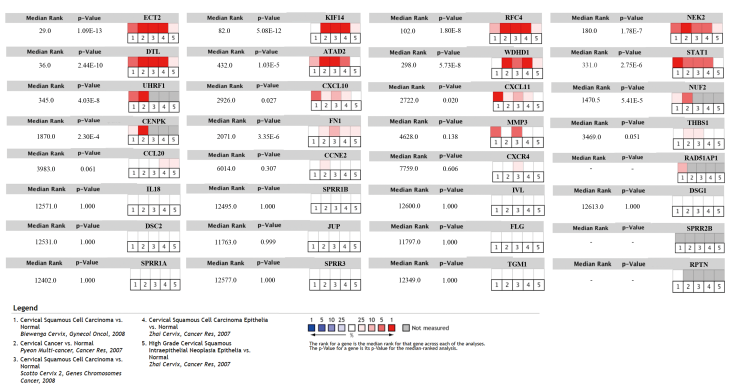


**FIGURE S2**


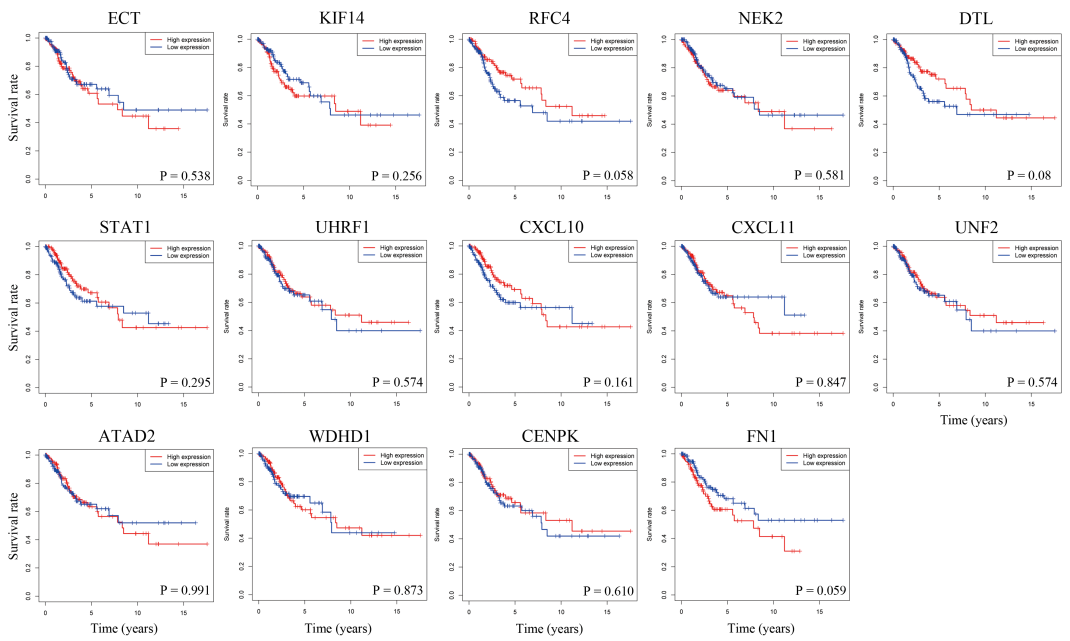


**FIGURE S3**


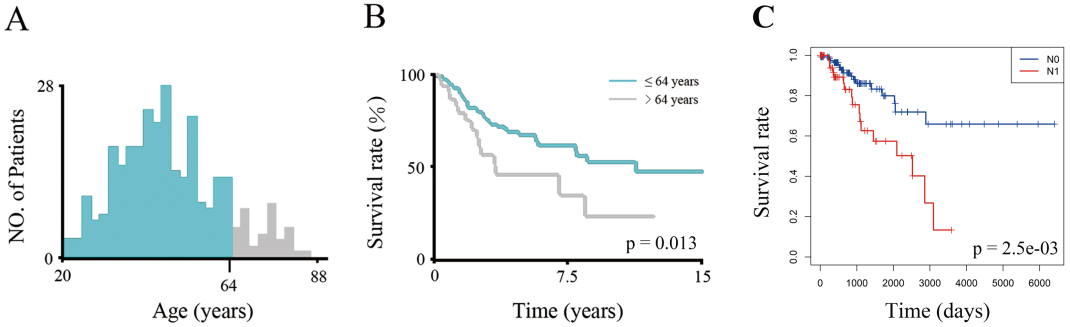


**FIGURE S4**


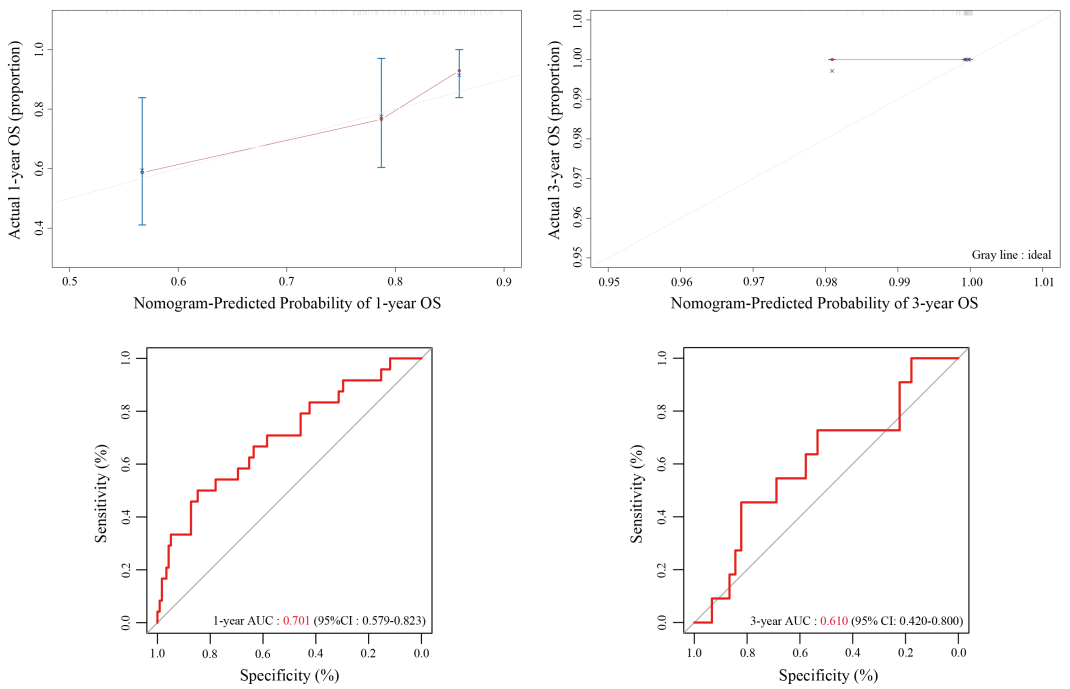


**FIGURE S5**

**Supplementary Tables**

| **Table SI. Antibodies for western blotting.** | | | | |
| --- | --- | --- | --- | --- |
| Antibodies | Molecular weight (kDa) | Dilution | Source | Catalog Number |
| DSG2 | 140 | 1:1000 | Proteintech, USA | 21880-1-AP |
| MMP1 | 60/55 | 1:1000 | Proteintech, USA | 10371-2-AP |
| SPP1 | 70 | 1:500 | Wanleibio, China | TA806784 |
| MCM2 | 110 | 1:500 | Proteintech, USA | 10513-1-AP |
| β-actin | 41 | 1:3000 | Proteintech, USA | 60008-1-lg |

| **Table SII. Clinical features of CC patients from GEO datasets.** | | | | | | | | |
| --- | --- | --- | --- | --- | --- | --- | --- | --- |
| **Series** | **GSM** | **Source** | **Age** | **Histology** | **Grade** | **Stage** | **LNM** | **Country** |
| GSE6791 | 155665 | cervical normal | 35 | N/A | N/A | N/A | N/A | USA |
| GSE6791 | 155666 | cervical normal | 44 | N/A | N/A | N/A | N/A | USA |
| GSE6791 | 155667 | cervical normal | 45 | N/A | N/A | N/A | N/A | USA |
| GSE6791 | 155668 | cervical normal | 66 | N/A | N/A | N/A | N/A | USA |
| GSE6791 | 155669 | cervical normal | 43 | N/A | N/A | N/A | N/A | USA |
| GSE6791 | 155670 | cervical normal | 56 | N/A | N/A | N/A | N/A | USA |
| GSE6791 | 155671 | cervical normal | 48 | N/A | N/A | N/A | N/A | USA |
| GSE6791 | 155672 | cervical normal | 43 | N/A | N/A | N/A | N/A | USA |
| [GSE63514](https://www.ncbi.nlm.nih.gov/geo/query/acc.cgi?acc=GSE63514) | 1551311 | cervical normal | N/A | N/A | N/A | N/A | N/A | USA |
| [GSE63514](https://www.ncbi.nlm.nih.gov/geo/query/acc.cgi?acc=GSE63514) | 1551312 | cervical normal | N/A | N/A | N/A | N/A | N/A | USA |
| [GSE63514](https://www.ncbi.nlm.nih.gov/geo/query/acc.cgi?acc=GSE63514) | 1551313 | cervical normal | N/A | N/A | N/A | N/A | N/A | USA |
| [GSE63514](https://www.ncbi.nlm.nih.gov/geo/query/acc.cgi?acc=GSE63514) | 1551314 | cervical normal | N/A | N/A | N/A | N/A | N/A | USA |
| [GSE63514](https://www.ncbi.nlm.nih.gov/geo/query/acc.cgi?acc=GSE63514) | 1551315 | cervical normal | N/A | N/A | N/A | N/A | N/A | USA |
| [GSE63514](https://www.ncbi.nlm.nih.gov/geo/query/acc.cgi?acc=GSE63514) | 1551316 | cervical normal | N/A | N/A | N/A | N/A | N/A | USA |
| [GSE63514](https://www.ncbi.nlm.nih.gov/geo/query/acc.cgi?acc=GSE63514) | 1551317 | cervical normal | N/A | N/A | N/A | N/A | N/A | USA |
| [GSE63514](https://www.ncbi.nlm.nih.gov/geo/query/acc.cgi?acc=GSE63514) | 1551318 | cervical normal | N/A | N/A | N/A | N/A | N/A | USA |
| [GSE63514](https://www.ncbi.nlm.nih.gov/geo/query/acc.cgi?acc=GSE63514) | 1551319 | cervical normal | N/A | N/A | N/A | N/A | N/A | USA |
| [GSE63514](https://www.ncbi.nlm.nih.gov/geo/query/acc.cgi?acc=GSE63514) | 1551320 | cervical normal | N/A | N/A | N/A | N/A | N/A | USA |
| [GSE63514](https://www.ncbi.nlm.nih.gov/geo/query/acc.cgi?acc=GSE63514) | 1551321 | cervical normal | N/A | N/A | N/A | N/A | N/A | USA |
| [GSE63514](https://www.ncbi.nlm.nih.gov/geo/query/acc.cgi?acc=GSE63514) | 1551322 | cervical normal | N/A | N/A | N/A | N/A | N/A | USA |
| [GSE63514](https://www.ncbi.nlm.nih.gov/geo/query/acc.cgi?acc=GSE63514) | 1551323 | cervical normal | N/A | N/A | N/A | N/A | N/A | USA |
| [GSE63514](https://www.ncbi.nlm.nih.gov/geo/query/acc.cgi?acc=GSE63514) | 1551324 | cervical normal | N/A | N/A | N/A | N/A | N/A | USA |
| [GSE63514](https://www.ncbi.nlm.nih.gov/geo/query/acc.cgi?acc=GSE63514) | 1551325 | cervical normal | N/A | N/A | N/A | N/A | N/A | USA |
| [GSE63514](https://www.ncbi.nlm.nih.gov/geo/query/acc.cgi?acc=GSE63514) | 1551326 | cervical normal | N/A | N/A | N/A | N/A | N/A | USA |
| [GSE63514](https://www.ncbi.nlm.nih.gov/geo/query/acc.cgi?acc=GSE63514) | 1551327 | cervical normal | N/A | N/A | N/A | N/A | N/A | USA |
| [GSE63514](https://www.ncbi.nlm.nih.gov/geo/query/acc.cgi?acc=GSE63514) | 1551328 | cervical normal | N/A | N/A | N/A | N/A | N/A | USA |
| [GSE63514](https://www.ncbi.nlm.nih.gov/geo/query/acc.cgi?acc=GSE63514) | 1551329 | cervical normal | N/A | N/A | N/A | N/A | N/A | USA |
| [GSE63514](https://www.ncbi.nlm.nih.gov/geo/query/acc.cgi?acc=GSE63514) | 1551330 | cervical normal | N/A | N/A | N/A | N/A | N/A | USA |
| [GSE63514](https://www.ncbi.nlm.nih.gov/geo/query/acc.cgi?acc=GSE63514) | 1551331 | cervical normal | N/A | N/A | N/A | N/A | N/A | USA |
| [GSE63514](https://www.ncbi.nlm.nih.gov/geo/query/acc.cgi?acc=GSE63514) | 1551332 | cervical normal | N/A | N/A | N/A | N/A | N/A | USA |
| [GSE63514](https://www.ncbi.nlm.nih.gov/geo/query/acc.cgi?acc=GSE63514) | 1551333 | cervical normal | N/A | N/A | N/A | N/A | N/A | USA |
| [GSE63514](https://www.ncbi.nlm.nih.gov/geo/query/acc.cgi?acc=GSE63514) | 1551334 | cervical normal | N/A | N/A | N/A | N/A | N/A | USA |
| GSE5787 | 135229 | cervical cancer | 31 | Squamous cell carcinoma | Moderately differentiated | IIB | N/A | Canada |
| GSE5787 | 135233 | cervical cancer | 47 | Squamous cell carcinoma | Moderately differentiated | IB | N/A | Canada |
| GSE5787 | 135235 | cervical cancer | 37 | Squamous cell carcinoma | Moderately differentiated | IB | N/A | Canada |
| GSE5787 | 135241 | cervical cancer | 61 | Squamous cell carcinoma | Well differentiated | IIIB | N/A | Canada |
| GSE5787 | 135242 | cervical cancer | 63 | Squamous cell carcinoma | Poorly differentiated | IIB | N/A | Canada |
| GSE5787 | 135244 | cervical cancer | 31 | Adenosquamous carcinoma | Moderately differentiated | IIIB | N/A | Canada |
| GSE5787 | 135247 | cervical cancer | 51 | Squamous cell carcinoma | Poorly differentiated | IVB | N/A | Canada |
| GSE5787 | 135248 | cervical cancer | 70 | Squamous cell carcinoma | Moderately differentiated | IIB | N/A | Canada |
| GSE5787 | 135249 | cervical cancer | 61 | Squamous cell carcinoma | Well differentiated | IIIB | N/A | Canada |
| GSE5787 | 135254 | cervical cancer | 42 | Squamous cell carcinoma | Moderately differentiated | IIIB | N/A | Canada |
| GSE5787 | 135256 | cervical cancer | 35 | Adenosquamous carcinoma | Moderately differentiated | IIB | N/A | Canada |
| GSE6791 | 155645 | cervical cancer | 35 | N/A | Well/moderately | II | N/A | USA |
| GSE6791 | 155646 | cervical cancer | 27 | N/A | Well/moderately | IB2 | N/A | USA |
| GSE6791 | 155647 | cervical cancer | 40 | N/A | Well/moderately | IB | N/A | USA |
| GSE6791 | 155648 | cervical cancer | 42 | N/A | Poorly/undiffer | IB | N/A | USA |
| GSE6791 | 155649 | cervical cancer | 44 | N/A | Poorly/undiffer | II/III | N/A | USA |
| GSE6791 | 155650 | cervical cancer | 41 | N/A | Poorly/undiffer | IB | N/A | USA |
| GSE6791 | 155651 | cervical cancer | 57 | N/A | Well/moderately | IB | N/A | USA |
| GSE6791 | 155652 | cervical cancer | 44 | N/A | Well/moderately | IV | N/A | USA |
| GSE6791 | 155653 | cervical cancer | 44 | N/A | Well/moderately | IB | N/A | USA |
| GSE6791 | 155654 | cervical cancer | 25 | N/A | Well/moderately | IB | N/A | USA |
| GSE6791 | 155655 | cervical cancer | 30 | N/A | Poorly/undiffer | IB2 | N/A | USA |
| GSE6791 | 155656 | cervical cancer | 47 | N/A | Poorly/undiffer | IB2 | N/A | USA |
| GSE6791 | 155657 | cervical cancer | 63 | N/A | Poorly/undiffer | IB | N/A | USA |
| GSE6791 | 155658 | cervical cancer | 38 | N/A | Poorly/undiffer | IB | N/A | USA |
| GSE6791 | 155659 | cervical cancer | 52 | N/A | Poorly/undiffer | IIIB | N/A | USA |
| GSE6791 | 155660 | cervical cancer | 38 | N/A | Poorly/undiffer | IB2 | N/A | USA |
| GSE6791 | 155661 | cervical cancer | 48 | N/A | Well/moderately | IB2 | N/A | USA |
| GSE6791 | 155662 | cervical cancer | 53 | N/A | Poorly/undiffer | IB | N/A | USA |
| GSE6791 | 155663 | cervical cancer | 55 | N/A | Poorly/undiffer | IB1 | N/A | USA |
| GSE6791 | 155664 | cervical cancer | 26 | N/A | Poorly/undiffer | IB2 | N/A | USA |
| GSE26511 | 651831 | cervical cancer | 56.4 | N/A | N/A | IB1 | negative | Netherlands |
| GSE26511 | 651832 | cervical cancer | 45.8 | N/A | N/A | IB1 | negative | Netherlands |
| GSE26511 | 651833 | cervical cancer | 49.5 | N/A | N/A | IB1 | negative | Netherlands |
| GSE26511 | 651834 | cervical cancer | 34.7 | N/A | N/A | IIA | negative | Netherlands |
| GSE26511 | 651835 | cervical cancer | 55.5 | N/A | N/A | IB1 | negative | Netherlands |
| GSE26511 | 651836 | cervical cancer | 38.5 | N/A | N/A | IB1 | negative | Netherlands |
| GSE26511 | 651837 | cervical cancer | 34.9 | N/A | N/A | IB1 | negative | Netherlands |
| GSE26511 | 651838 | cervical cancer | 47.4 | N/A | N/A | IB1 | negative | Netherlands |
| GSE26511 | 651839 | cervical cancer | 42.3 | N/A | N/A | IB1 | negative | Netherlands |
| GSE26511 | 651840 | cervical cancer | 35.8 | N/A | N/A | IB2 | negative | Netherlands |
| GSE26511 | 651841 | cervical cancer | 51.6 | N/A | N/A | IIA | negative | Netherlands |
| GSE26511 | 651842 | cervical cancer | 42 | N/A | N/A | IB2 | negative | Netherlands |
| GSE26511 | 651843 | cervical cancer | 71 | N/A | N/A | IB2 | negative | Netherlands |
| GSE26511 | 651844 | cervical cancer | 35.9 | N/A | N/A | IB2 | negative | Netherlands |
| GSE26511 | 651845 | cervical cancer | 68.9 | N/A | N/A | IIA | negative | Netherlands |
| GSE26511 | 651846 | cervical cancer | 47.4 | N/A | N/A | IB2 | negative | Netherlands |
| GSE26511 | 651847 | cervical cancer | 31.5 | N/A | N/A | IB1 | negative | Netherlands |
| GSE26511 | 651848 | cervical cancer | 72.7 | N/A | N/A | IIA | negative | Netherlands |
| GSE26511 | 651849 | cervical cancer | 39.9 | N/A | N/A | IB1 | negative | Netherlands |
| GSE26511 | 651850 | cervical cancer | 50.7 | N/A | N/A | IB1 | negative | Netherlands |
| GSE26511 | 651851 | cervical cancer | 56.2 | N/A | N/A | IB1 | Positive | Netherlands |
| GSE26511 | 651852 | cervical cancer | 29.1 | N/A | N/A | IB1 | Positive | Netherlands |
| GSE26511 | 651853 | cervical cancer | 32.2 | N/A | N/A | IIA | Positive | Netherlands |
| GSE26511 | 651854 | cervical cancer | 60.6 | N/A | N/A | IB1 | Positive | Netherlands |
| GSE26511 | 651855 | cervical cancer | 49.9 | N/A | N/A | IIA | Positive | Netherlands |
| GSE26511 | 651856 | cervical cancer | 34.9 | N/A | N/A | IB2 | Positive | Netherlands |
| GSE26511 | 651857 | cervical cancer | 32.7 | N/A | N/A | IB2 | Positive | Netherlands |
| GSE26511 | 651858 | cervical cancer | 40.4 | N/A | N/A | IB1 | Positive | Netherlands |
| GSE26511 | 651859 | cervical cancer | 48.5 | N/A | N/A | IB2 | Positive | Netherlands |
| GSE26511 | 651860 | cervical cancer | 37.4 | N/A | N/A | IB1 | Positive | Netherlands |
| GSE26511 | 651861 | cervical cancer | 37 | N/A | N/A | IB2 | Positive | Netherlands |
| GSE26511 | 651862 | cervical cancer | 32 | N/A | N/A | IB1 | Positive | Netherlands |
| GSE26511 | 651863 | cervical cancer | 37.4 | N/A | N/A | IB1 | Positive | Netherlands |
| GSE26511 | 651864 | cervical cancer | 45.5 | N/A | N/A | IB2 | Positive | Netherlands |
| GSE26511 | 651865 | cervical cancer | 72.5 | N/A | N/A | IB1 | Positive | Netherlands |
| GSE26511 | 651866 | cervical cancer | 42.3 | N/A | N/A | IB1 | Positive | Netherlands |
| GSE26511 | 651867 | cervical cancer | 46.3 | N/A | N/A | IB1 | Positive | Netherlands |
| GSE26511 | 651868 | cervical cancer | 34.2 | N/A | N/A | IB2 | Positive | Netherlands |
| GSE26511 | 651869 | cervical cancer | 50.5 | N/A | N/A | IIA | Positive | Netherlands |
| GSE63514 | 1551411 | cervical cancer | N/A | Squamous cell carcinoma | N/A | N/A | N/A | USA |
| GSE63514 | 1551412 | cervical cancer | N/A | Squamous cell carcinoma | N/A | N/A | N/A | USA |
| GSE63514 | 1551413 | cervical cancer | N/A | Squamous cell carcinoma | N/A | N/A | N/A | USA |
| GSE63514 | 1551414 | cervical cancer | N/A | Squamous cell carcinoma | N/A | N/A | N/A | USA |
| GSE63514 | 1551415 | cervical cancer | N/A | Squamous cell carcinoma | N/A | N/A | N/A | USA |
| GSE63514 | 1551416 | cervical cancer | N/A | Squamous cell carcinoma | N/A | N/A | N/A | USA |
| GSE63514 | 1551417 | cervical cancer | N/A | Squamous cell carcinoma | N/A | N/A | N/A | USA |
| GSE63514 | 1551418 | cervical cancer | N/A | Squamous cell carcinoma | N/A | N/A | N/A | USA |
| GSE63514 | 1551419 | cervical cancer | N/A | Squamous cell carcinoma | N/A | N/A | N/A | USA |
| GSE63514 | 1551420 | cervical cancer | N/A | Squamous cell carcinoma | N/A | N/A | N/A | USA |
| GSE63514 | 1551421 | cervical cancer | N/A | Squamous cell carcinoma | N/A | N/A | N/A | USA |
| GSE63514 | 1551422 | cervical cancer | N/A | Squamous cell carcinoma | N/A | N/A | N/A | USA |
| GSE63514 | 1551423 | cervical cancer | N/A | Squamous cell carcinoma | N/A | N/A | N/A | USA |
| GSE63514 | 1551424 | cervical cancer | N/A | Squamous cell carcinoma | N/A | N/A | N/A | USA |
| GSE63514 | 1551425 | cervical cancer | N/A | Squamous cell carcinoma | N/A | N/A | N/A | USA |
| GSE63514 | 1551426 | cervical cancer | N/A | Squamous cell carcinoma | N/A | N/A | N/A | USA |
| GSE63514 | 1551427 | cervical cancer | N/A | Squamous cell carcinoma | N/A | N/A | N/A | USA |
| GSE63514 | 1551428 | cervical cancer | N/A | Squamous cell carcinoma | N/A | N/A | N/A | USA |
| GSE63514 | 1551429 | cervical cancer | N/A | Squamous cell carcinoma | N/A | N/A | N/A | USA |
| GSE63514 | 1551430 | cervical cancer | N/A | Squamous cell carcinoma | N/A | N/A | N/A | USA |
| GSE63514 | 1551431 | cervical cancer | N/A | Squamous cell carcinoma | N/A | N/A | N/A | USA |
| GSE63514 | 1551432 | cervical cancer | N/A | Squamous cell carcinoma | N/A | N/A | N/A | USA |
| GSE63514 | 1551433 | cervical cancer | N/A | Squamous cell carcinoma | N/A | N/A | N/A | USA |
| GSE63514 | 1551434 | cervical cancer | N/A | Squamous cell carcinoma | N/A | N/A | N/A | USA |
| GSE63514 | 1551435 | cervical cancer | N/A | Squamous cell carcinoma | N/A | N/A | N/A | USA |
| GSE63514 | 1551436 | cervical cancer | N/A | Squamous cell carcinoma | N/A | N/A | N/A | USA |
| GSE63514 | 1551437 | cervical cancer | N/A | Squamous cell carcinoma | N/A | N/A | N/A | USA |
| GSE63514 | 1551438 | cervical cancer | N/A | Squamous cell carcinoma | N/A | N/A | N/A | USA |

*LNM: lymph node metastasis*

| **Table SIII.** **Differentially Expressed Genes.** | | | | | | |
| --- | --- | --- | --- | --- | --- | --- |
| **Gene** | **logFC** | **AveExpr** | **t** | **P.Value** | **adj.P.Val** | **B** |
| CRNN | -3.642596987 | 8.121107297 | 7.098961366 | 3.89859E-10 | 1.0658E-06 | 12.7830619 |
| SPINK7 | -3.485702096 | 7.584416456 | 5.650731011 | 2.18504E-07 | 6.0749E-05 | 6.84023637 |
| TMPRSS11B | -3.387722918 | 6.70532651 | 5.544984447 | 3.4013E-07 | 7.6529E-05 | 6.42561627 |
| CRISP3 | -3.329690101 | 5.773377166 | 5.027989521 | 2.81029E-06 | 0.00026356 | 4.45091156 |
| KRTDAP | -3.128060827 | 9.004127181 | 4.401322791 | 3.17512E-05 | 0.00106634 | 2.19563986 |
| MAL | -3.059431835 | 9.024312043 | 5.207602541 | 1.36338E-06 | 0.00017336 | 5.12646629 |
| CRCT1 | -2.937793241 | 8.018030289 | 4.811780075 | 6.60624E-06 | 0.00043685 | 3.65405623 |
| DSG1 | -2.913454826 | 6.958001196 | 4.575707195 | 1.64415E-05 | 0.00073454 | 2.80607746 |
| SPRR3 | -2.874565762 | 11.15202515 | 3.894432955 | 0.000198178 | 0.00306123 | 0.50654593 |
| CNFN | -2.784564958 | 9.167257564 | 5.311225163 | 8.93613E-07 | 0.00014 | 5.52145412 |
| SBSN | -2.784005746 | 8.825701176 | 5.18601668 | 1.48811E-06 | 0.00018704 | 5.04465821 |
| ALOX12 | -2.700233504 | 7.718692732 | 7.159035654 | 2.97414E-10 | 1.0163E-06 | 13.0373548 |
| SPINK5 | -2.681507516 | 10.5230951 | 5.0903339 | 2.18921E-06 | 0.00022992 | 4.68405325 |
| KRT13 | -2.677946342 | 11.73042676 | 3.361646488 | 0.001172903 | 0.00857676 | -1.1140731 |
| KRT1 | -2.669628332 | 9.253425969 | 4.40243672 | 3.16193E-05 | 0.00106387 | 2.19949413 |
| KRT4 | -2.619945307 | 10.59142352 | 3.953180997 | 0.000161345 | 0.00272437 | 0.69537224 |
| ENDOU | -2.4259059 | 6.628912968 | 7.708383336 | 2.45619E-11 | 1.9185E-07 | 15.3795036 |
| DAPL1 | -2.42022266 | 9.361175529 | 4.371814018 | 3.54434E-05 | 0.00113525 | 2.09375358 |
| PPP1R3C | -2.414678661 | 8.350052548 | 4.988868967 | 3.28468E-06 | 0.00028643 | 4.30536929 |
| LCE3D | -2.396382303 | 8.67718113 | 4.552514703 | 1.79594E-05 | 0.00076694 | 2.7240863 |
| IL1R2 | -2.341850655 | 8.248109995 | 5.421421089 | 5.67984E-07 | 0.00010679 | 5.94548653 |
| BBOX1 | -2.282140333 | 7.094228051 | 4.783098927 | 7.38921E-06 | 0.00046814 | 3.54976562 |
| SCEL | -2.280909003 | 8.203633648 | 3.870598253 | 0.000215309 | 0.0032085 | 0.4304838 |
| RHCG | -2.271678242 | 10.44699216 | 4.204271622 | 6.56801E-05 | 0.00162638 | 1.52333209 |
| SCEL | -2.224265153 | 7.176646854 | 4.146712026 | 8.09318E-05 | 0.00182925 | 1.33061298 |
| DMKN | -2.211775567 | 8.635997354 | 5.081271037 | 2.27035E-06 | 0.00023377 | 4.65007157 |
| RPTN | -2.194339769 | 5.949671435 | 4.462940582 | 2.52024E-05 | 0.00092754 | 2.40972292 |
| CWH43 | -2.192404609 | 7.727636943 | 4.51050889 | 2.10615E-05 | 0.00083445 | 2.57620649 |
| SOSTDC1 | -2.180054868 | 6.754774667 | 5.638854843 | 2.29676E-07 | 6.2788E-05 | 6.79350576 |
| SPRR1A | -2.156428132 | 10.83794309 | 3.677276299 | 0.000417039 | 0.00466673 | -0.1744803 |
| EDN3 | -2.128648743 | 5.558426178 | 7.629088528 | 3.52688E-11 | 2.1426E-07 | 15.0398644 |
| CWH43 | -2.105663792 | 5.908873071 | 4.160590202 | 7.69695E-05 | 0.00177192 | 1.37692393 |
| KLK11 | -2.096277966 | 9.061051229 | 4.686723693 | 1.07395E-05 | 0.00057342 | 3.20185883 |
| CLIC3 | -2.090192451 | 7.584385523 | 5.299349151 | 9.38116E-07 | 0.00014155 | 5.47599763 |
| TMPRSS11E | -2.060650985 | 8.666521894 | 3.396905867 | 0.001047975 | 0.0079767 | -1.0121964 |
| SPRR3 | -2.050989981 | 11.37851122 | 3.177580815 | 0.00208579 | 0.01227695 | -1.632762 |
| TGM3 | -2.029549244 | 7.015552512 | 5.059278702 | 2.47967E-06 | 0.00024805 | 4.56773748 |
| SPRR2B | -2.020702041 | 10.11941646 | 3.202803029 | 0.001929959 | 0.01170239 | -1.5630117 |
| IL1R2 | -2.008585586 | 7.651679426 | 5.175747605 | 1.5513E-06 | 0.0001904 | 5.00579781 |
| ESRG | -2.008249264 | 6.082759298 | 4.043583574 | 0.00011717 | 0.0022613 | 0.98962403 |
| TPRG1 | -2.007281138 | 7.763105574 | 4.953347154 | 3.78256E-06 | 0.00031128 | 4.17372699 |
| SPRR1A | -1.983437902 | 11.50071555 | 2.725002676 | 0.007838576 | 0.02901653 | -2.8092371 |
| RDH12 | -1.982567649 | 8.049326886 | 7.166603956 | 2.87434E-10 | 1.0163E-06 | 13.0694213 |
| GREB1 | -1.970828697 | 5.328136006 | 4.384356993 | 3.38259E-05 | 0.0011048 | 2.13701034 |
| IVL | -1.942724587 | 9.934756969 | 4.461768411 | 2.53138E-05 | 0.00092784 | 2.40563371 |
| BNIPL | -1.938619147 | 7.651750255 | 5.172205766 | 1.57369E-06 | 0.0001912 | 4.99240348 |
| UPK1A | -1.928124524 | 6.709509744 | 8.600120248 | 4.09186E-13 | 7.4574E-09 | 19.2181363 |
| EREG | -1.917263592 | 6.083142223 | 3.339862474 | 0.001256951 | 0.0089202 | -1.1766156 |
| SFTA2 | -1.879016323 | 5.823488258 | 4.891940795 | 4.82222E-06 | 0.00036018 | 3.94732584 |
| KRT78 | -1.876114287 | 7.700084119 | 5.567283442 | 3.09913E-07 | 7.2412E-05 | 6.51276874 |
| HOPX | -1.868932254 | 10.08311388 | 5.239603429 | 1.19709E-06 | 0.00015868 | 5.24804909 |
| TMPRSS11D | -1.835286191 | 9.968278671 | 3.202194569 | 0.001933586 | 0.011714 | -1.5646994 |
| ALOX12B | -1.82815205 | 7.394371571 | 4.938565592 | 4.01078E-06 | 0.00032091 | 4.11909223 |
| CRYAB | -1.823808541 | 8.786301431 | 4.413953106 | 3.02871E-05 | 0.00104206 | 2.23937612 |
| SPRR1B | -1.814254336 | 11.1871069 | 2.831012558 | 0.005818331 | 0.0237525 | -2.5467761 |
| FLG | -1.80750346 | 5.326348164 | 3.443161964 | 0.00090302 | 0.00732281 | -0.8773455 |
| CXCL14 | -1.798764616 | 9.14874506 | 3.075291687 | 0.002846137 | 0.01501182 | -1.9112244 |
| NSG1 | -1.786096137 | 8.348677502 | 4.780160237 | 7.47435E-06 | 0.00047135 | 3.53909925 |
| KLK13 | -1.780200975 | 8.261427793 | 3.686737318 | 0.000403955 | 0.00459166 | -0.1453796 |
| CYSRT1 | -1.77725767 | 8.186468393 | 5.447200481 | 5.1056E-07 | 0.00010078 | 6.04525878 |
| HPGD | -1.776495717 | 7.310370951 | 3.419421546 | 0.000974875 | 0.00763191 | -0.9467256 |
| VSIG10L | -1.762677057 | 8.704913393 | 4.354920808 | 3.77405E-05 | 0.00117703 | 2.03561386 |
| CLCA4 | -1.755979956 | 8.859847596 | 2.738248788 | 0.007555154 | 0.02829883 | -2.7768944 |
| A2ML1 | -1.710317382 | 8.31350541 | 3.799411195 | 0.000275305 | 0.00365851 | 0.20521591 |
| GYS2 | -1.710218742 | 4.43207173 | 6.40712198 | 8.4589E-09 | 9.8402E-06 | 9.89180591 |
| KLK12 | -1.699347841 | 8.258709924 | 4.568672693 | 1.68883E-05 | 0.00074585 | 2.78118316 |
| GJB6 | -1.698204243 | 10.17590423 | 2.557274978 | 0.012366719 | 0.03974783 | -3.2073966 |
| THSD4 | -1.696938259 | 8.722571442 | 6.888087804 | 1.00419E-09 | 2.2877E-06 | 11.8940556 |
| ESR1 | -1.686166839 | 9.702168377 | 3.998206238 | 0.000137652 | 0.00247164 | 0.84137283 |
| LGALS7 | -1.685580082 | 11.76339166 | 2.998178066 | 0.003581847 | 0.01734853 | -2.1163985 |
| PRSS27 | -1.66477142 | 6.647869661 | 5.179828045 | 1.52588E-06 | 0.00018893 | 5.02123456 |
| TGM1 | -1.661248697 | 9.402088212 | 3.53445201 | 0.000670649 | 0.00612763 | -0.6072659 |
| AKR1B10 | -1.653254187 | 10.67112542 | 3.022846111 | 0.003329246 | 0.01658501 | -2.0512146 |
| DSC2 | -1.64872215 | 8.419036827 | 3.644316679 | 0.000465838 | 0.00496329 | -0.2754441 |
| THSD4 | -1.645064965 | 9.16806132 | 5.958771515 | 5.91294E-08 | 3.0663E-05 | 8.06604159 |
| PRSS3 | -1.64169331 | 8.171545357 | 5.124885145 | 1.90507E-06 | 0.00021546 | 4.81388347 |
| CRABP2 | -1.638899137 | 10.74269532 | 4.534055046 | 1.92639E-05 | 0.00079889 | 2.65900076 |
| CXCL14 | -1.636750655 | 10.30125716 | 3.214708907 | 0.001860244 | 0.01141733 | -1.5299389 |
| ALOX15B | -1.636077159 | 7.855492585 | 4.995425371 | 3.20005E-06 | 0.00028371 | 4.32972047 |
| C10orf99 | -1.632279904 | 9.749941011 | 2.5680658 | 0.012016236 | 0.03897186 | -3.1824227 |
| ZBED2 | -1.613687381 | 7.712986346 | 3.84525707 | 0.000235071 | 0.00337336 | 0.34996387 |
| SLURP1 | -1.610094257 | 6.881011877 | 5.579493275 | 2.94501E-07 | 7.1356E-05 | 6.56055308 |
| CYP2B6 | -1.599612947 | 7.777403581 | 4.720968235 | 9.40749E-06 | 0.00053604 | 3.32502666 |
| TMEM40 | -1.592358748 | 9.401911577 | 5.707169711 | 1.72308E-07 | 5.3226E-05 | 7.06287098 |
| KRT78 | -1.581028938 | 6.406090713 | 4.951097633 | 3.81645E-06 | 0.00031219 | 4.16540691 |
| KLK10 | -1.576322159 | 7.351959917 | 4.944593429 | 3.91614E-06 | 0.00031685 | 4.14136163 |
| IL18 | -1.575843564 | 8.067456105 | 4.317076896 | 4.34203E-05 | 0.0012702 | 1.90587223 |
| FMO2 | -1.574000909 | 7.299050599 | 4.082201345 | 0.000102073 | 0.00209192 | 1.11665681 |
| TTC22 | -1.572986342 | 9.897451758 | 5.200287172 | 1.40446E-06 | 0.00017734 | 5.0987233 |
| CRISP2 | -1.571013354 | 4.506212206 | 7.372693083 | 1.13173E-10 | 5.3865E-07 | 13.9449972 |
| EMP1 | -1.57051673 | 7.537515441 | 5.234039167 | 1.22451E-06 | 0.00016055 | 5.22688266 |
| LOC100130476 | -1.568805612 | 5.709662235 | 5.854776306 | 9.21911E-08 | 3.8773E-05 | 7.6493535 |
| GJB2 | -1.563613217 | 11.13505286 | 3.369503503 | 0.001143908 | 0.00842332 | -1.0914405 |
| S100A12 | -1.550116238 | 8.523596288 | 3.100501641 | 0.002637875 | 0.01427979 | -1.8432576 |
| LOC441178 | -1.547882203 | 7.722893284 | 8.919167215 | 9.3995E-14 | 2.5696E-09 | 20.5939839 |
| GBP6 | -1.539707594 | 9.226936701 | 3.522115454 | 0.000698358 | 0.00628006 | -0.6440658 |
| KRT15 | -1.536749179 | 11.65101009 | 2.89525878 | 0.004839134 | 0.02106696 | -2.3837319 |
| KLK8 | -1.533387848 | 8.728403472 | 4.041613615 | 0.000117995 | 0.00226981 | 0.98316511 |
| LY6D | -1.530972357 | 11.00068914 | 3.14565105 | 0.002299926 | 0.01304174 | -1.7204478 |
| HPGD | -1.517134481 | 8.250450015 | 3.05572566 | 0.003018198 | 0.01558386 | -1.9636736 |
| KLK10 | -1.504037156 | 9.993064091 | 3.073823527 | 0.002858724 | 0.01504918 | -1.9151692 |
| IGK | 1.502160818 | 9.426585487 | -3.220420353 | 0.001827643 | 0.01129408 | -1.5140398 |
| C1QC | 1.506763725 | 9.687251702 | -5.780474888 | 1.26398E-07 | 4.5466E-05 | 7.35338621 |
| KIF14 | 1.511482421 | 8.691748824 | -6.523078234 | 5.07987E-09 | 7.1216E-06 | 10.3708549 |
| RFC4 | 1.511812338 | 10.29404714 | -6.243212922 | 1.73111E-08 | 1.4378E-05 | 9.2191943 |
| RAD51AP1 | 1.518472259 | 9.679437331 | -5.917676719 | 7.04969E-08 | 3.2225E-05 | 7.90105037 |
| RSAD2 | 1.520006664 | 7.986104113 | -3.547824139 | 0.00064179 | 0.00598599 | -0.5672712 |
| DSG2 | 1.521194847 | 11.23415419 | -6.014671091 | 4.65188E-08 | 2.5953E-05 | 8.29115138 |
| NUF2 | 1.528161537 | 9.099495295 | -5.916912868 | 7.07274E-08 | 3.2225E-05 | 7.89798767 |
| CENPK | 1.528774793 | 8.622123418 | -5.814085613 | 1.09603E-07 | 4.3168E-05 | 7.4870846 |
| UCP2 | 1.536713425 | 9.859283019 | -5.620115716 | 2.48455E-07 | 6.601E-05 | 6.71985482 |
| CXCL10 | 1.538556546 | 10.48973959 | -3.319950568 | 0.001338699 | 0.00930148 | -1.2335149 |
| COL12A1 | 1.539407772 | 10.33504922 | -4.506619681 | 2.13737E-05 | 0.00084376 | 2.56255548 |
| UHRF1 | 1.540031776 | 10.31374204 | -6.241886255 | 1.74113E-08 | 1.4378E-05 | 9.21377316 |
| WDHD1 | 1.540458674 | 7.746168753 | -7.12533108 | 3.46207E-10 | 1.0658E-06 | 12.8946315 |
| FST | 1.54153141 | 8.186042474 | -2.821332449 | 0.005980722 | 0.02419504 | -2.5710835 |
| BIRC3 | 1.543156228 | 8.151370137 | -3.619200223 | 0.000506615 | 0.00520075 | -0.3519467 |
| SNX10 | 1.551770839 | 8.559572932 | -5.4248488 | 5.59998E-07 | 0.00010668 | 5.95874024 |
| IGLC1 | 1.5560432 | 12.35117128 | -4.016035035 | 0.000129225 | 0.00237972 | 0.89948892 |
| FNDC3B | 1.559615693 | 7.213039017 | -5.904717646 | 7.45096E-08 | 3.2823E-05 | 7.84911045 |
| ALCAM | 1.561184793 | 10.74811058 | -4.274061533 | 5.08795E-05 | 0.00138676 | 1.75925313 |
| MS4A7 | 1.567050443 | 8.61547604 | -5.122940122 | 1.92006E-06 | 0.00021556 | 4.80656317 |
| GREM1 | 1.571531338 | 7.436974186 | -3.884417826 | 0.000205211 | 0.00312533 | 0.47454661 |
| RGS1 | 1.574061411 | 10.55750517 | -5.307936819 | 9.05724E-07 | 0.00014101 | 5.5088629 |
| NEK2 | 1.575249552 | 9.522978495 | -6.549356093 | 4.52385E-09 | 6.509E-06 | 10.4797644 |
| STAT1 | 1.577312738 | 8.742543992 | -5.419600479 | 5.7227E-07 | 0.00010715 | 5.93844844 |
| MICB | 1.581286842 | 8.607293838 | -4.523951045 | 2.00163E-05 | 0.00081186 | 2.62344147 |
| TCAM1P | 1.582728355 | 6.937811921 | -4.554926348 | 1.77955E-05 | 0.00076311 | 2.73260075 |
| FAM72A | 1.58716739 | 9.289721483 | -6.643458551 | 2.98379E-09 | 4.5316E-06 | 10.87078 |
| ADAMDEC1 | 1.589709379 | 8.572658157 | -4.251336035 | 5.53049E-05 | 0.00146233 | 1.68216268 |
| MMP3 | 1.596711136 | 8.57608986 | -3.307101628 | 0.001394073 | 0.00953477 | -1.2700947 |
| DTL | 1.599017357 | 8.546029323 | -7.121503276 | 3.52228E-10 | 1.0658E-06 | 12.878431 |
| IGKV1-17 | 1.599333222 | 9.478446649 | -3.240325151 | 0.001718156 | 0.010879 | -1.4584608 |
| DTL | 1.600028239 | 10.04113454 | -6.663799442 | 2.72652E-09 | 4.3845E-06 | 10.955501 |
| CCNE2 | 1.605222915 | 9.388302736 | -5.711656529 | 1.69078E-07 | 5.3226E-05 | 7.08060934 |
| HELLS | 1.606393391 | 8.460126824 | -5.924658229 | 6.84241E-08 | 3.1704E-05 | 7.92905011 |
| C1QB | 1.609795521 | 9.34497022 | -5.371699366 | 6.97194E-07 | 0.00011987 | 5.75366074 |
| ADAM12 | 1.627300792 | 7.509794921 | -4.33804083 | 4.01791E-05 | 0.00121942 | 1.97765755 |
| EPCAM | 1.630787116 | 10.80512675 | -4.412251022 | 3.04805E-05 | 0.00104419 | 2.23347773 |
| THBS1 | 1.63225759 | 8.50020656 | -4.582726851 | 1.60072E-05 | 0.00072498 | 2.83094137 |
| PRTFDC1 | 1.638813066 | 7.714236678 | -4.693650603 | 1.0456E-05 | 0.0005649 | 3.2267323 |
| FAM46C | 1.651457626 | 8.63453198 | -4.227756033 | 6.02882E-05 | 0.00154464 | 1.60244658 |
| APOBEC3B | 1.657482525 | 9.034696025 | -4.986967644 | 3.30962E-06 | 0.00028723 | 4.29831068 |
| GBP5 | 1.663744272 | 7.403340692 | -3.866425611 | 0.000218449 | 0.00323738 | 0.41720065 |
| COL12A1 | 1.66708465 | 7.672213222 | -3.937624491 | 0.000170403 | 0.00280626 | 0.64518554 |
| MOCOS | 1.681975696 | 8.545871989 | -4.872406198 | 5.2079E-06 | 0.00037466 | 3.87561802 |
| FYB | 1.683226097 | 7.931437767 | -4.637803738 | 1.29648E-05 | 0.00064257 | 3.02678756 |
| PLOD2 | 1.687790227 | 10.11866846 | -5.902656443 | 7.51683E-08 | 3.2823E-05 | 7.84085314 |
| CFH | 1.691808955 | 8.985789882 | -4.263777238 | 5.28382E-05 | 0.00142172 | 1.72433441 |
| JUP | 1.697431214 | 11.99495728 | -4.355411045 | 3.76718E-05 | 0.00117563 | 2.03729912 |
| ATAD2 | 1.698297051 | 8.843414961 | -6.839078099 | 1.24999E-09 | 2.6286E-06 | 11.6883144 |
| ABCA17P | 1.702678744 | 7.075230207 | -3.606000185 | 0.000529382 | 0.00534218 | -0.3920008 |
| ECT2 | 1.707137209 | 10.39102458 | -6.656046432 | 2.82188E-09 | 4.4082E-06 | 10.923201 |
| PLSCR1 | 1.715624399 | 10.12658911 | -7.230745408 | 2.15173E-10 | 8.8639E-07 | 13.3414448 |
| IGH | 1.72049515 | 11.47431053 | -3.411050324 | 0.001001473 | 0.00776531 | -0.971105 |
| CPNE8 | 1.732903015 | 7.546231898 | -4.810306007 | 6.64444E-06 | 0.00043717 | 3.64868791 |
| E2F7 | 1.734743545 | 9.116931707 | -5.911968551 | 7.22374E-08 | 3.2641E-05 | 7.87816673 |
| IGLC1 | 1.74057915 | 12.51051492 | -4.231310275 | 5.95103E-05 | 0.00153405 | 1.6144444 |
| SAMSN1 | 1.74919067 | 8.076975485 | -5.537612771 | 3.50742E-07 | 7.8273E-05 | 6.39683851 |
| KRT7 | 1.753613314 | 8.456287264 | -2.762568297 | 0.007059027 | 0.02703694 | -2.7171762 |
| SIX1 | 1.75608451 | 7.638364436 | -4.220485966 | 6.191E-05 | 0.00156986 | 1.57792532 |
| KIF14 | 1.762835143 | 7.850286731 | -6.338730446 | 1.14126E-08 | 1.1773E-05 | 9.61048603 |
| IGK | 1.768717309 | 10.75806667 | -3.552352231 | 0.000632287 | 0.00593074 | -0.5537032 |
| ALCAM | 1.769728856 | 8.08650086 | -4.530304925 | 1.95399E-05 | 0.00080196 | 2.64579742 |
| FAM26F | 1.788439108 | 8.416655022 | -5.548714426 | 3.34881E-07 | 7.5742E-05 | 6.44018381 |
| VCAM1 | 1.789309514 | 8.96723754 | -3.941434461 | 0.000168141 | 0.0027934 | 0.65746456 |
| FN1 | 1.798274072 | 11.05303139 | -4.968938595 | 3.55556E-06 | 0.00030073 | 4.23144781 |
| RGS1 | 1.800974705 | 8.452054681 | -5.065705738 | 2.41662E-06 | 0.00024355 | 4.59178007 |
| CCL20 | 1.818703736 | 8.85493712 | -3.861742678 | 0.000222027 | 0.00326833 | 0.4023047 |
| IGJ | 1.821194418 | 9.709214588 | -4.007377491 | 0.000133254 | 0.0024256 | 0.87124673 |
| FN1 | 1.826066792 | 11.00374473 | -5.052896339 | 2.54386E-06 | 0.00025106 | 4.54387736 |
| LOC101928916 | 1.842751851 | 9.673400692 | -4.608145834 | 1.45256E-05 | 0.00068347 | 2.92116069 |
| IFI44 | 1.84492691 | 9.592091084 | -4.608439568 | 1.45093E-05 | 0.00068329 | 2.92220492 |
| CCDC71L | 1.845482202 | 8.402875277 | -6.162965829 | 2.45281E-08 | 1.7646E-05 | 8.89197546 |
| CXCL11 | 1.849234271 | 7.904020516 | -2.847261897 | 0.005554808 | 0.02304103 | -2.50582 |
| MLF1 | 1.851621617 | 7.922105921 | -4.752116554 | 8.33645E-06 | 0.00050543 | 3.43749279 |
| MS4A4A | 1.873658387 | 7.272524651 | -5.438737462 | 5.28753E-07 | 0.00010252 | 6.01248139 |
| NCEH1 | 1.88374375 | 7.996248379 | -5.387950635 | 6.52074E-07 | 0.00011613 | 5.81626896 |
| FAM26F | 1.890110408 | 9.664263857 | -5.945632198 | 6.25519E-08 | 3.0663E-05 | 8.01324209 |
| COL8A1 | 1.893407214 | 7.577018963 | -4.397621874 | 3.2193E-05 | 0.00107605 | 2.18283871 |
| LOX | 1.898776989 | 8.298703171 | -3.968379514 | 0.000152941 | 0.00264291 | 0.74453209 |
| ANKRD36BP2 | 1.918850186 | 5.604104092 | -3.946699574 | 0.000165061 | 0.00276408 | 0.67444643 |
| CXCR4 | 1.934343085 | 10.04937916 | -5.120145854 | 1.9418E-06 | 0.00021756 | 4.79604907 |
| RARRES1 | 1.935762017 | 8.119633895 | -3.728066662 | 0.000351241 | 0.00423931 | -0.0176384 |
| GREM1 | 1.94937359 | 8.046251455 | -4.385478929 | 3.36847E-05 | 0.00110216 | 2.14088322 |
| IGHG1 | 1.955843454 | 12.62950405 | -4.578007345 | 1.6298E-05 | 0.0007316 | 2.81422225 |
| ZIC2 | 1.993076059 | 7.342081065 | -4.765551548 | 7.91201E-06 | 0.0004877 | 3.4861286 |
| MMP10 | 1.993369856 | 7.775087504 | -3.172514281 | 0.002118478 | 0.01240786 | -1.6467217 |
| FN1 | 2.009953231 | 10.57287312 | -5.273707389 | 1.04174E-06 | 0.00014642 | 5.37801564 |
| CXCL1 | 2.064061951 | 9.569193369 | -3.892188467 | 0.000199734 | 0.00307705 | 0.49936971 |
| KLHDC7B | 2.089862573 | 9.303528143 | -3.873854057 | 0.000212888 | 0.00319226 | 0.44085509 |
| FN1 | 2.12368981 | 10.96581492 | -5.378559098 | 6.77787E-07 | 0.00011881 | 5.78007734 |
| CXCL11 | 2.14990452 | 7.624455011 | -3.234917538 | 0.001747277 | 0.01098296 | -1.4735862 |
| CMPK2 | 2.234878156 | 9.050200231 | -5.488684621 | 4.299E-07 | 9.0403E-05 | 6.20625698 |
| MMP12 | 2.29576667 | 10.08362463 | -4.70271365 | 1.00962E-05 | 0.00055367 | 3.2593075 |
| IFI44L | 2.304255153 | 8.568462093 | -4.456433519 | 2.58268E-05 | 0.00094249 | 2.38703067 |
| ELAVL2 | 2.319800788 | 7.645328556 | -4.914259863 | 4.41559E-06 | 0.00034051 | 4.02944191 |
| HS6ST2 | 2.37158132 | 8.17523858 | -3.81576844 | 0.000260249 | 0.00355313 | 0.25672289 |
| MCM2 | 2.381114325 | 11.15363911 | -9.870999528 | 1.17567E-15 | 6.428E-11 | 24.6795024 |
| CXCL8 | 2.441521026 | 10.34624294 | -5.760574947 | 1.3751E-07 | 4.7888E-05 | 7.27437296 |
| HS6ST2 | 2.489800253 | 7.246240534 | -4.033198462 | 0.000121582 | 0.00230768 | 0.95559763 |
| INHBA | 2.542719912 | 8.936000671 | -5.88502982 | 8.10406E-08 | 3.4348E-05 | 7.77028472 |
| SPP1 | 2.57347128 | 9.962361713 | -5.417072939 | 5.78275E-07 | 0.00010791 | 5.92867928 |
| SYCP2 | 2.795402815 | 7.015550311 | -5.09652655 | 2.1354E-06 | 0.00022759 | 4.70729045 |
| CDKN2A | 2.85044401 | 9.457668266 | -6.486397859 | 5.97077E-09 | 7.8508E-06 | 10.2190433 |
| MMP1 | 2.897723146 | 8.536906492 | -4.191124111 | 6.88985E-05 | 0.00167126 | 1.47916274 |
| CTHRC1 | 3.004094463 | 9.80545838 | -6.923633872 | 8.56535E-10 | 2.2301E-06 | 12.0434925 |

*The mRNAs expressed in CC. vs. control. CC: Cervical Cancer*

| **Table SIV. Clinical features of CC patients from the TCGA-CC cohort.** | | | | | | | | | | |
| --- | --- | --- | --- | --- | --- | --- | --- | --- | --- | --- |
| **Id** | **Number** | **Futime** | **Fustat** | **Age** | **Race** | **Grade** | **Stage** | **T** | **M** | **N** |
| TCGA-2W-A8YY | 49 | 533 | 0 | 51 | white | G3 | IB2 | T2a2 | M0 | N0 |
| TCGA-4J-AA1J | 222 | 406 | 0 | 31 | white | G3 | IB2 | T1b2 | M0 | N0 |
| TCGA-BI-A0VR | 332 | 1505 | 0 | 53 | white | G3 | IIIB | T2b | M0 | N1 |
| TCGA-BI-A0VS | 20 | 1735 | 0 | 48 | white | G3 | IB | T1b1 | M0 | N0 |
| TCGA-BI-A20A | 887 | 462 | 0 | 49 | white | G3 | IB1 | T1b1 | M0 | N0 |
| TCGA-C5-A0TN | 726 | 348 | 1 | 21 | black or african american | G3 | IB2 | T1b | MX | N1 |
| TCGA-C5-A1BE | 704 | 2094 | 1 | 64 | white | G2 | IB2 | T1b | MX | N1 |
| TCGA-C5-A1BF | 391 | 570 | 1 | 46 | white | G1 | IB | TX | MX | N0 |
| TCGA-C5-A1BI | 64 | 1112 | 0 | 31 | white | G2 | IIIB | unknow | unknow | unknow |
| TCGA-C5-A1BJ | 448 | 4385 | 0 | 34 | white | G2 | IIB | unknow | unknow | unknow |
| TCGA-C5-A1BK | 236 | 5385 | 0 | 36 | black or african american | G2 | IB | T1b | MX | N0 |
| TCGA-C5-A1BL | 747 | 5271 | 0 | 32 | white | G2 | IB | IB | unknow | unknow |
| TCGA-C5-A1BM | 966 | 2520 | 1 | 78 | black or african american | G2 | IB | T2 | MX | N1 |
| TCGA-C5-A1BN | 723 | 166 | 1 | 26 | white | G3 | IB2 | unknow | unknow | unknow |
| TCGA-C5-A1BQ | 768 | 604 | 1 | 65 | white | G2 | IIIB | unknow | unknow | unknow |
| TCGA-C5-A1M5 | 159 | 2052 | 1 | 53 | white | G2 | IB | T1b | M0 | N0 |
| TCGA-C5-A1M6 | 573 | 955 | 1 | 55 | white | G3 | IIB | unknow | unknow | unknow |
| TCGA-C5-A1M7 | 443 | 1409 | 0 | 37 | white | G2 | IB | T1b | MX | N0 |
| TCGA-C5-A1M8 | 651 | 919 | 0 | 43 | white | G2 | IB1 | T1b | MX | N0 |
| TCGA-C5-A1M9 | 797 | 1065 | 1 | 46 | white | G3 | IB1 | T3b | M0 | N1 |
| TCGA-C5-A1ME | 285 | 1756 | 0 | 40 | white | G1 | IB1 | T1b1 | M0 | N0 |
| TCGA-C5-A1MF | 302 | 1617 | 0 | 49 | white | G2 | IB2 | T1b2 | MX | N0 |
| TCGA-C5-A1MH | 110 | 1186 | 1 | 71 | white | G3 | IIIB | unknow | unknow | unknow |
| TCGA-C5-A1MI | 464 | 1083 | 1 | 50 | white | G2 | IB1 | T1b1 | M0 | N1 |
| TCGA-C5-A1MJ | 593 | 14 | 1 | 61 | white | G2 | IB1 | T1b1 | MX | N0 |
| TCGA-C5-A1MK | 203 | 74 | 1 | 79 | white | G3 | unknow | unknow | unknow | unknow |
| TCGA-C5-A1ML | 30 | 636 | 1 | 49 | white | G3 | IB2 | unknow | unknow | unknow |
| TCGA-C5-A1MN | 151 | 1245 | 1 | 42 | white | G2 | IIIB | unknow | unknow | unknow |
| TCGA-C5-A1MP | 539 | 109 | 0 | 34 | black or african american | G3 | IB2 | T1b2 | MX | N1 |
| TCGA-C5-A1MQ | 94 | 1031 | 0 | 35 | white | G2 | IIA | T2b | M0 | N0 |
| TCGA-C5-A2LS | 949 | 1345 | 0 | 37 | white | G1 | IB2 | T1b2 | M0 | N0 |
| TCGA-C5-A2LT | 998 | 2226 | 0 | 38 | white | G3 | IB | T1b | M0 | N0 |
| TCGA-C5-A2LV | 978 | 2234 | 0 | 36 | black or african american | G3 | IB | T1b | MX | N1 |
| TCGA-C5-A2LX | 676 | 2526 | 0 | 54 | white | G2 | IB1 | T2 | MX | N1 |
| TCGA-C5-A2LY | 316 | 2383 | 0 | 30 | white | G2 | IB1 | T1b1 | M0 | N0 |
| TCGA-C5-A2LZ | 702 | 3046 | 1 | 65 | white | G2 | IIIB | unknow | unknow | unknow |
| TCGA-C5-A2M1 | 947 | 1169 | 0 | 37 | white | G2 | IB1 | T1b1 | MX | N0 |
| TCGA-C5-A2M2 | 209 | 1011 | 1 | 56 | white | G2 | IB2 | unknow | unknow | unknow |
| TCGA-C5-A3HD | 366 | 1582 | 0 | 51 | white | G2 | IIB | unknow | unknow | unknow |
| TCGA-C5-A3HE | 169 | 548 | 0 | 44 | white | G3 | IB2 | T1b2 | M0 | N0 |
| TCGA-C5-A3HF | 766 | 543 | 1 | 24 | white | G2 | IB2 | unknow | unknow | unknow |
| TCGA-C5-A3HL | 717 | 621 | 0 | 76 | white | G2 | IB2 | T1b2 | MX | N0 |
| TCGA-C5-A7CG | 368 | 6408 | 0 | 55 | white | G2 | IB | T1b | MX | N0 |
| TCGA-C5-A7CH | 238 | 4694 | 0 | 43 | white | G2 | IIB | unknow | unknow | unknow |
| TCGA-C5-A7CJ | 639 | 3097 | 1 | 42 | white | G2 | IIA | T2a | MX | N1 |
| TCGA-C5-A7CK | 236 | 4086 | 1 | 58 | white | G2 | IVA | unknow | unknow | unknow |
| TCGA-C5-A7CL | 200 | 471 | 1 | 48 | white | G2 | IIIB | unknow | unknow | unknow |
| TCGA-C5-A7CM | 565 | 619 | 0 | 35 | white | G2 | IB2 | unknow | unknow | unknow |
| TCGA-C5-A7CO | 863 | 4482 | 0 | 68 | white | G2 | IB2 | unknow | unknow | unknow |
| TCGA-C5-A7UC | 329 | 523 | 1 | 48 | white | G3 | IB | T1b | M0 | N0 |
| TCGA-C5-A7UE | 682 | 4738 | 0 | 45 | white | G2 | IB1 | unknow | unknow | unknow |
| TCGA-C5-A7UH | 188 | 3988 | 0 | 55 | white | G3 | IIIB | unknow | unknow | unknow |
| TCGA-C5-A7UI | 361 | 2888 | 1 | 42 | black or african american | G3 | IB1 | T1b1 | MX | N0 |
| TCGA-C5-A7X3 | 773 | 284 | 1 | 70 | white | G2 | IIIB | unknow | unknow | unknow |
| TCGA-C5-A7X5 | 921 | 414 | 1 | 72 | black or african american | G3 | IVB | unknow | unknow | unknow |
| TCGA-C5-A7X8 | 937 | 83 | 0 | 35 | white | G2 | IB1 | T2a | M0 | N1 |
| TCGA-C5-A7XC | 921 | 1551 | 0 | 26 | white | G2 | IB1 | T1b | M0 | N0 |
| TCGA-C5-A8XH | 599 | 1394 | 1 | 39 | white | unknow | IB1 | T1b1 | MX | N0 |
| TCGA-C5-A8XI | 631 | 254 | 0 | 69 | black or african american | G3 | IB2 | T1b2 | MX | N0 |
| TCGA-C5-A8XJ | 244 | 4467 | 0 | 74 | white | unknow | IB | unknow | unknow | unknow |
| TCGA-C5-A8XK | 582 | 3039 | 0 | 30 | black or african american | G3 | unknow | unknow | unknow | unknow |
| TCGA-C5-A8YQ | 695 | 715 | 1 | 79 | white | G2 | IB1 | unknow | unknow | unknow |
| TCGA-C5-A8YR | 28 | 837 | 1 | 56 | white | G3 | IB | T1b | M0 | N0 |
| TCGA-C5-A8YT | 386 | 633 | 1 | 36 | white | G3 | IB1 | unknow | unknow | unknow |
| TCGA-C5-A8ZZ | 778 | 636 | 0 | 41 | white | G2 | IIB | unknow | unknow | unknow |
| TCGA-C5-A901 | 366 | 518 | 0 | 44 | white | G2 | unknow | TX | MX | N1 |
| TCGA-C5-A902 | 520 | 149 | 0 | 35 | black or african american | G3 | IB2 | unknow | unknow | unknow |
| TCGA-C5-A905 | 794 | 4879 | 0 | 37 | black or african american | G2 | IB | T1b | MX | N0 |
| TCGA-C5-A907 | 726 | 361 | 0 | 47 | white | G2 | IB2 | unknow | unknow | unknow |
| TCGA-DG-A2KH | 698 | 34 | 0 | 25 | not reported | GX | IB1 | T1b1 | M0 | N0 |
| TCGA-DG-A2KJ | 652 | 1791 | 0 | 50 | not reported | G3 | IIIB | T1b | M0 | N1 |
| TCGA-DG-A2KK | 630 | 1544 | 0 | 51 | not reported | G3 | IIIB | T1b1 | M0 | N1 |
| TCGA-DG-A2KL | 99 | 1367 | 0 | 53 | not reported | G1 | IIA | T2a | M0 | N0 |
| TCGA-DG-A2KM | 776 | 1218 | 0 | 46 | not reported | G2 | IB1 | T1b1 | M0 | N1 |
| TCGA-DR-A0ZL | 813 | 2669 | 0 | 53 | asian | G3 | IB | T1b1 | M0 | N0 |
| TCGA-DR-A0ZM | 901 | 1791 | 0 | 61 | white | G2 | IIIB | T2b | M0 | N0 |
| TCGA-DS-A0VK | 194 | 1118 | 1 | 45 | white | G3 | IB | T1b1 | M0 | N1 |
| TCGA-DS-A0VL | 479 | 1692 | 1 | 25 | white | G2 | IB | T1b1 | M0 | N0 |
| TCGA-DS-A0VM | 97 | 3589 | 0 | 51 | white | G3 | IB | T1b1 | M0 | N1 |
| TCGA-DS-A0VN | 327 | 3609 | 0 | 47 | white | G2 | IB | T1b2 | M0 | N0 |
| TCGA-DS-A1O9 | 541 | 266 | 1 | 75 | white | G3 | IVA | T4 | M0 | N0 |
| TCGA-DS-A1OA | 479 | 879 | 1 | 77 | asian | G3 | IB | T1b1 | M0 | N1 |
| TCGA-DS-A1OB | 326 | 861 | 1 | 45 | white | G2 | IB | T1b2 | M0 | N1 |
| TCGA-DS-A1OC | 129 | 376 | 0 | 47 | white | G2 | IB | T1b1 | M0 | N1 |
| TCGA-DS-A1OD | 37 | 3874 | 0 | 49 | white | G3 | IB1 | T1b1 | M0 | N0 |
| TCGA-DS-A3LQ | 658 | 699 | 0 | 46 | not reported | G3 | IIIB | T3b | M0 | N1 |
| TCGA-DS-A5RQ | 764 | 208 | 0 | 80 | white | G2 | IB1 | T1b1 | MX | N0 |
| TCGA-DS-A7WF | 747 | 492 | 1 | 41 | not reported | G3 | IB2 | T1b2 | MX | N0 |
| TCGA-DS-A7WH | 738 | 533 | 0 | 34 | white | G2 | IB1 | T1b1 | MX | N0 |
| TCGA-DS-A7WI | 111 | 252 | 1 | 43 | white | G2 | IIA2 | T2a2 | MX | N1 |
| TCGA-EA-A1QS | 702 | 1203 | 0 | 46 | white | G2 | IB1 | T1b1 | M0 | N0 |
| TCGA-EA-A1QT | 344 | 1243 | 0 | 47 | white | G2 | IB | T1b | M0 | N0 |
| TCGA-EA-A3HQ | 315 | 1136 | 0 | 60 | white | G2 | II | T2a | M0 | N0 |
| TCGA-EA-A3HR | 600 | 940 | 0 | 57 | white | G2 | II | T2a | M0 | N0 |
| TCGA-EA-A3HS | 187 | 959 | 0 | 35 | white | G1 | IB | T1b | M0 | N0 |
| TCGA-EA-A3HT | 472 | 954 | 0 | 68 | white | G1 | IB | T1b | M0 | N0 |
| TCGA-EA-A3HU | 106 | 1013 | 0 | 43 | white | G2 | II | T2a2 | M0 | N0 |
| TCGA-EA-A3QD | 412 | 397 | 0 | 59 | white | G3 | IIIB | T1b1 | M0 | N1 |
| TCGA-EA-A3QE | 716 | 761 | 0 | 45 | white | G2 | IB | T1b | M0 | N0 |
| TCGA-EA-A3Y4 | 231 | 441 | 0 | 40 | white | G3 | IB | T1b | M0 | N0 |
| TCGA-EA-A410 | 150 | 803 | 0 | 51 | white | G2 | IIA2 | T2a2 | M0 | N0 |
| TCGA-EA-A411 | 941 | 747 | 0 | 50 | white | G2 | IB1 | T1b1 | M0 | N0 |
| TCGA-EA-A439 | 63 | 441 | 0 | 50 | asian | G3 | IIA1 | T2a1 | M0 | N0 |
| TCGA-EA-A43B | 495 | 791 | 0 | 43 | white | G2 | IB1 | T1b1 | M0 | N0 |
| TCGA-EA-A44S | 529 | 369 | 0 | 31 | white | G2 | IIIB | T2a2 | M0 | N1 |
| TCGA-EA-A4BA | 369 | 390 | 0 | 49 | white | G2 | IB2 | T1b2 | M0 | N0 |
| TCGA-EA-A50E | 935 | 227 | 1 | 45 | asian | G2 | IVA | T4 | M0 | N1 |
| TCGA-EA-A556 | 413 | 453 | 0 | 38 | white | G3 | IB1 | T1b1 | M0 | N0 |
| TCGA-EA-A5FO | 193 | 427 | 0 | 59 | white | G2 | IB1 | T1b1 | M0 | N0 |
| TCGA-EA-A5O9 | 459 | 423 | 0 | 39 | white | G2 | IB2 | T1b2 | M0 | N0 |
| TCGA-EA-A5ZD | 322 | 457 | 0 | 40 | asian | G2 | IB1 | T1b1 | M0 | N0 |
| TCGA-EA-A5ZE | 763 | 456 | 0 | 54 | asian | G3 | IB1 | T1b1 | M0 | N0 |
| TCGA-EA-A5ZF | 160 | 455 | 0 | 56 | asian | G2 | IB1 | T1b1 | M0 | NX |
| TCGA-EA-A6QX | 587 | 413 | 0 | 49 | asian | G3 | IIIB | T1b1 | M0 | N1 |
| TCGA-EA-A78R | 71 | 410 | 0 | 54 | asian | G2 | IB1 | T1b1 | M0 | NX |
| TCGA-EA-A97N | 648 | 11 | 0 | 38 | white | G2 | IB2 | T1b2 | M0 | N0 |
| TCGA-EK-A2GZ | 854 | 383 | 0 | 64 | black or african american | G2 | IIIB | unknow | unknow | unknow |
| TCGA-EK-A2H0 | 496 | 1847 | 0 | 24 | white | G3 | IIB | unknow | unknow | unknow |
| TCGA-EK-A2H1 | 478 | 799 | 0 | 20 | white | G3 | IB2 | unknow | unknow | unknow |
| TCGA-EK-A2IP | 182 | 202 | 0 | 28 | white | G3 | IB1 | T1b1 | MX | N0 |
| TCGA-EK-A2IR | 185 | 3442 | 0 | 48 | white | G3 | IB2 | T1b | MX | N0 |
| TCGA-EK-A2PG | 551 | 46 | 0 | 88 | black or african american | G3 | unknow | unknow | unknow | unknow |
| TCGA-EK-A2PI | 968 | 42 | 0 | 44 | white | G2 | IIIB | unknow | unknow | unknow |
| TCGA-EK-A2PK | 363 | 12 | 0 | 43 | white | G3 | IB1 | unknow | unknow | unknow |
| TCGA-EK-A2PL | 337 | 13 | 0 | 36 | white | unknow | IIIB | unknow | unknow | unknow |
| TCGA-EK-A2PM | 918 | 18 | 0 | 81 | white | G3 | IIB | unknow | unknow | unknow |
| TCGA-EK-A2R7 | 3 | 27 | 0 | 45 | white | G3 | IB | T1b1 | M0 | N1 |
| TCGA-EK-A2R8 | 455 | 44 | 0 | 48 | white | G3 | IB2 | T1b2 | MX | N1 |
| TCGA-EK-A2R9 | 898 | 4 | 0 | 58 | white | G3 | IB1 | T1b1 | unknow | N0 |
| TCGA-EK-A2RA | 539 | 489 | 0 | 74 | not reported | G3 | IIA2 | T2a2 | MX | N0 |
| TCGA-EK-A2RB | 440 | 9 | 0 | 48 | white | G3 | IVB | unknow | unknow | unknow |
| TCGA-EK-A2RC | 846 | 129 | 0 | 33 | not reported | G3 | IB1 | unknow | unknow | unknow |
| TCGA-EK-A2RE | 360 | 57 | 0 | 26 | white | G2 | IIA | T2a2 | MX | N0 |
| TCGA-EK-A2RJ | 603 | 53 | 0 | 51 | white | G3 | IB2 | T1b2 | MX | N0 |
| TCGA-EK-A2RK | 79 | 13 | 0 | 67 | white | G3 | IA2 | T1b1 | MX | N0 |
| TCGA-EK-A2RL | 99 | 1453 | 1 | 32 | black or african american | G2 | IB | T1b1 | MX | N1 |
| TCGA-EK-A2RM | 842 | 50 | 0 | 40 | white | G3 | IB | unknow | unknow | unknow |
| TCGA-EK-A2RN | 18 | 71 | 0 | 45 | white | G2 | IB1 | T2b | MX | N1 |
| TCGA-EK-A2RO | 828 | 2 | 0 | 59 | american indian | G1 | IIB | unknow | unknow | unknow |
| TCGA-EK-A3GJ | 54 | 3 | 0 | 51 | native hawaiian | G3 | IB1 | T1b1 | unknow | N0 |
| TCGA-EK-A3GK | 876 | 15 | 0 | 33 | white | G3 | IB1 | T1b1 | MX | N0 |
| TCGA-EK-A3GM | 601 | 0 | 0 | 65 | white | G2 | IIA | unknow | unknow | unknow |
| TCGA-EK-A3GN | 272 | 27 | 0 | 47 | white | G3 | IIIB | TX | unknow | NX |
| TCGA-EX-A1H5 | 679 | 619 | 0 | 58 | black or african american | G3 | IIB | T2b | MX | N1 |
| TCGA-EX-A1H6 | 843 | 241 | 0 | 38 | white | G1 | IB1 | T1b1 | MX | N0 |
| TCGA-EX-A3L1 | 999 | 463 | 0 | 32 | white | G3 | IIA1 | T2a1 | M0 | N0 |
| TCGA-EX-A449 | 977 | 447 | 0 | 42 | white | G1 | IVB | T1b1 | M1 | NX |
| TCGA-EX-A69L | 475 | 602 | 0 | 41 | asian | G3 | IB1 | T1b1 | M0 | N0 |
| TCGA-EX-A69M | 224 | 253 | 1 | 62 | white | G3 | IB2 | T1b2 | M0 | N0 |
| TCGA-EX-A8YF | 362 | 473 | 0 | 44 | white | G3 | IB1 | T2a1 | M0 | N0 |
| TCGA-FU-A23K | 974 | 372 | 0 | 28 | white | G3 | IIIB | T1b1 | MX | N1 |
| TCGA-FU-A23L | 565 | 725 | 0 | 60 | white | G3 | IIA1 | T2a1 | MX | N0 |
| TCGA-FU-A2QG | 315 | 579 | 0 | 29 | white | G2 | IB1 | T1b1 | unknow | N0 |
| TCGA-FU-A3EO | 88 | 490 | 0 | 55 | white | G2 | IIB | T2b | MX | N0 |
| TCGA-FU-A3HY | 211 | 954 | 0 | 47 | white | G2 | IB2 | T1b2 | MX | N1 |
| TCGA-FU-A3HZ | 295 | 1103 | 0 | 64 | white | G3 | IIA2 | T2a2 | M0 | N0 |
| TCGA-FU-A3NI | 4 | 638 | 1 | 45 | white | G2 | IB1 | T1b1 | MX | N1 |
| TCGA-FU-A3TQ | 845 | 795 | 0 | 55 | white | G2 | IIIB | T2b | MX | N1 |
| TCGA-FU-A3TX | 241 | 45 | 0 | 78 | not reported | G3 | IB2 | T1b2 | M0 | N0 |
| TCGA-FU-A3WB | 473 | 491 | 0 | 43 | white | G2 | IIA2 | T2a2 | MX | N0 |
| TCGA-FU-A3YQ | 335 | 861 | 0 | 35 | white | G1 | IB1 | T1b1 | MX | N0 |
| TCGA-FU-A40J | 393 | 426 | 0 | 38 | white | G3 | IIIB | T2a1 | MX | N1 |
| TCGA-FU-A57G | 810 | 1078 | 0 | 49 | white | G2 | IB2 | T1b2 | MX | N0 |
| TCGA-FU-A5XV | 724 | 321 | 0 | 32 | white | G3 | IIIB | T1b2 | MX | N1 |
| TCGA-FU-A770 | 213 | 34 | 0 | 33 | white | G2 | IIIB | T2a2 | MX | N1 |
| TCGA-GH-A9DA | 177 | 540 | 0 | 27 | white | G3 | IB1 | T1b | MX | N0 |
| TCGA-HG-A2PA | 690 | 773 | 0 | 38 | white | G2 | IB2 | T1b2 | M0 | N0 |
| TCGA-HM-A3JJ | 457 | 659 | 1 | 40 | black or african american | G3 | IB1 | T1b1 | MX | N1 |
| TCGA-HM-A3JK | 186 | 351 | 0 | 64 | white | G3 | IIA2 | T2b | MX | N1 |
| TCGA-HM-A4S6 | 457 | 454 | 0 | 51 | white | G3 | IIIB | T1b2 | MX | N1 |
| TCGA-HM-A6W2 | 692 | 287 | 0 | 34 | black or african american | G3 | IVB | T2a | M1 | N0 |
| TCGA-IR-A3L7 | 758 | 4483 | 0 | 37 | white | G3 | IB1 | T1b1 | M0 | N0 |
| TCGA-IR-A3LA | 27 | 4078 | 0 | 60 | white | G3 | IB1 | T1b1 | M0 | N0 |
| TCGA-IR-A3LB | 409 | 2032 | 1 | 53 | white | G3 | IB1 | T1b1 | M0 | N0 |
| TCGA-IR-A3LC | 473 | 3571 | 0 | 40 | white | G3 | IB1 | T1b1 | M0 | N0 |
| TCGA-IR-A3LF | 764 | 2949 | 0 | 64 | white | G2 | IB1 | T1b1 | M0 | N0 |
| TCGA-IR-A3LH | 270 | 2394 | 0 | 49 | black or african american | G4 | IIA1 | T2a1 | M0 | N0 |
| TCGA-IR-A3LI | 797 | 2493 | 0 | 48 | white | G2 | IVB | T2b | M1 | N1 |
| TCGA-IR-A3LK | 129 | 908 | 1 | 69 | white | G3 | IB2 | T1b2 | M0 | N0 |
| TCGA-IR-A3LL | 969 | 957 | 0 | 60 | white | G2 | IB1 | T1b1 | M0 | N0 |
| TCGA-JW-A5VG | 204 | 834 | 0 | 35 | american indian or alaska native | G3 | IIA | T2a | MX | NX |
| TCGA-JW-A5VH | 373 | 100 | 1 | 53 | american indian or alaska native | G2 | IVB | T4 | M1 | NX |
| TCGA-JW-A5VI | 873 | 747 | 0 | 45 | american indian or alaska native | G3 | IIB | T2b | MX | NX |
| TCGA-JW-A5VJ | 32 | 588 | 0 | 56 | american indian or alaska native | G3 | IIB | T2b | MX | NX |
| TCGA-JW-A5VK | 238 | 623 | 0 | 43 | black or african american | G3 | IB2 | T1b2 | MX | NX |
| TCGA-JW-A5VL | 930 | 474 | 0 | 37 | american indian or alaska native | G1 | IB2 | T1b2 | MX | NX |
| TCGA-JW-A69B | 27 | 863 | 0 | 44 | white | GX | IB2 | T1b2 | MX | NX |
| TCGA-JW-A852 | 629 | 252 | 1 | 42 | white | G2 | IIB | T2b | MX | N1 |
| TCGA-JW-AAVH | 203 | 552 | 0 | 46 | white | G2 | IB1 | T1b1 | M0 | N0 |
| TCGA-JX-A3PZ | 220 | 642 | 1 | 25 | white | G2 | IB | T1b | M0 | N0 |
| TCGA-JX-A3Q0 | 827 | 5957 | 0 | 63 | black or african american | G2 | III | T3 | M0 | N0 |
| TCGA-JX-A3Q8 | 905 | 922 | 0 | 40 | asian | G3 | IB1 | T1b1 | MX | N0 |
| TCGA-JX-A5QV | 964 | 243 | 0 | 37 | white | G3 | IB1 | T1b1 | M0 | N0 |
| TCGA-LP-A4AU | 479 | 343 | 0 | 35 | asian | G3 | IIIB | T2 | M0 | N1 |
| TCGA-LP-A4AV | 74 | 0 | 0 | 63 | asian | G2 | IB | T1b | M0 | N0 |
| TCGA-LP-A4AW | 289 | 27 | 0 | 52 | asian | G1 | IA | T1b | M0 | N0 |
| TCGA-LP-A4AX | 880 | 380 | 0 | 45 | asian | G3 | IB1 | T1b1 | M0 | N0 |
| TCGA-LP-A5U2 | 536 | 9 | 0 | 30 | asian | G3 | IIIB | T2a | MX | N1 |
| TCGA-LP-A5U3 | 958 | 25 | 0 | 40 | asian | G3 | IB1 | T1b1 | M0 | N0 |
| TCGA-LP-A7HU | 30 | 406 | 0 | 53 | asian | G3 | II | T2 | M0 | N0 |
| TCGA-MA-AA3W | 394 | 685 | 0 | 54 | white | G3 | IB1 | T1b1 | M0 | N1 |
| TCGA-MA-AA3X | 835 | 617 | 0 | 50 | black or african american | G2 | IIIB | T3b | MX | NX |
| TCGA-MA-AA3Y | 814 | 542 | 0 | 48 | white | G3 | IB1 | T1b1 | M0 | N0 |
| TCGA-MA-AA3Z | 205 | 595 | 0 | 43 | white | GX | IB2 | T1b2 | M0 | N0 |
| TCGA-MA-AA41 | 552 | 279 | 0 | 33 | white | G2 | IIB | T2 | M1 | N1 |
| TCGA-MA-AA42 | 566 | 259 | 0 | 75 | white | G3 | IIB | T2b | M0 | N0 |
| TCGA-MA-AA43 | 527 | 346 | 0 | 48 | white | G3 | IIIB | T3b | MX | NX |
| TCGA-MU-A51Y | 945 | 854 | 0 | 27 | white | G2 | IIA1 | T2a1 | MX | N0 |
| TCGA-MU-A5YI | 709 | 1053 | 0 | 60 | black or african american | G2 | IA1 | T1a1 | MX | NX |
| TCGA-MU-A8JM | 408 | 607 | 0 | 46 | white | G2 | IB1 | T1b | MX | N0 |
| TCGA-MY-A5BD | 725 | 1667 | 0 | 62 | white | G1 | IIB | T2b | M0 | N0 |
| TCGA-MY-A5BE | 765 | 1066 | 0 | 42 | black or african american | G3 | IB1 | T1b1 | MX | N0 |
| TCGA-MY-A5BF | 151 | 634 | 0 | 68 | white | G1 | IIA2 | T2a2 | MX | N0 |
| TCGA-MY-A913 | 285 | 524 | 0 | 28 | black or african american | G3 | IIA | Tis | M1 | N0 |
| TCGA-PN-A8MA | 138 | 90 | 0 | 43 | black or african american | G3 | IIB | T2b | M0 | N0 |
| TCGA-Q1-A5R1 | 361 | 474 | 0 | 32 | white | G2 | IB1 | T1b1 | MX | N0 |
| TCGA-Q1-A5R2 | 551 | 499 | 0 | 64 | white | G3 | unknow | TX | MX | NX |
| TCGA-Q1-A5R3 | 85 | 485 | 0 | 56 | white | G2 | unknow | TX | MX | NX |
| TCGA-Q1-A6DT | 220 | 275 | 1 | 55 | white | GX | I | TX | MX | NX |
| TCGA-Q1-A6DV | 439 | 491 | 0 | 36 | asian | G2 | IB1 | T1b1 | M0 | N0 |
| TCGA-Q1-A6DW | 881 | 534 | 0 | 44 | white | GX | I | TX | MX | NX |
| TCGA-Q1-A73O | 244 | 428 | 0 | 37 | not reported | GX | IB2 | TX | MX | NX |
| TCGA-Q1-A73P | 555 | 483 | 0 | 45 | white | G1 | IB1 | T1b | MX | N0 |
| TCGA-Q1-A73Q | 610 | 284 | 0 | 46 | white | GX | I | TX | MX | NX |
| TCGA-Q1-A73R | 12 | 567 | 0 | 45 | white | GX | I | TX | MX | NX |
| TCGA-Q1-A73S | 120 | 688 | 0 | 33 | white | G2 | IB1 | T1b1 | MX | N1 |
| TCGA-R2-A69V | 416 | 596 | 0 | 42 | white | G3 | IB | T1b | M0 | N0 |
| TCGA-RA-A741 | 634 | 444 | 0 | 34 | white | GX | IIB | unknow | unknow | unknow |
| TCGA-UC-A7PD | 260 | 355 | 1 | 21 | american indian or alaska native | G2 | IB | T1b | M0 | N0 |
| TCGA-UC-A7PF | 719 | 2859 | 1 | 44 | not reported | G2 | IB1 | T1b1 | M0 | N1 |
| TCGA-UC-A7PG | 850 | 370 | 1 | 44 | white | G1 | IIIB | T3b | MX | N1 |
| TCGA-UC-A7PI | 708 | 2050 | 0 | 44 | white | G1 | IB1 | T1b1 | MX | N0 |
| TCGA-VS-A8EB | 225 | 305 | 1 | 41 | white | GX | IIIB | T3b | M1 | NX |
| TCGA-VS-A8EC | 389 | 1232 | 0 | 55 | white | G2 | IIIB | T3b | M0 | NX |
| TCGA-VS-A8EG | 290 | 1149 | 0 | 36 | not reported | GX | IB1 | T1b1 | M0 | NX |
| TCGA-VS-A8EH | 938 | 804 | 0 | 56 | not reported | G2 | IIIB | T3b | M0 | NX |
| TCGA-VS-A8EI | 791 | 549 | 0 | 38 | white | GX | IIB | T3b | M0 | NX |
| TCGA-VS-A8EJ | 282 | 607 | 1 | 60 | white | G3 | IIB | T2b | M1 | NX |
| TCGA-VS-A8EK | 11 | 829 | 1 | 65 | white | G2 | IVA | T4 | M0 | NX |
| TCGA-VS-A8EL | 446 | 1810 | 0 | 38 | black or african american | G3 | IIB | T2b | M0 | NX |
| TCGA-VS-A8Q8 | 220 | 978 | 1 | 26 | white | G2 | IB | T1b | MX | N0 |
| TCGA-VS-A8Q9 | 823 | 1630 | 0 | 79 | white | G2 | IB1 | TX | MX | NX |
| TCGA-VS-A8QA | 233 | 1099 | 0 | 44 | not reported | GX | IB1 | T1b1 | MX | N0 |
| TCGA-VS-A8QC | 94 | 350 | 1 | 51 | white | G2 | IVA | TX | MX | NX |
| TCGA-VS-A8QF | 567 | 1617 | 0 | 42 | white | G2 | IIB | TX | MX | NX |
| TCGA-VS-A8QH | 8 | 1210 | 1 | 76 | white | G2 | IB1 | TX | MX | NX |
| TCGA-VS-A8QM | 287 | 951 | 1 | 47 | not reported | GX | IVB | TX | M1 | NX |
| TCGA-VS-A94W | 579 | 1103 | 0 | 39 | white | G2 | IIB | T2b | MX | N1 |
| TCGA-VS-A94X | 553 | 506 | 1 | 40 | black or african american | G2 | IIB | T2b | MX | NX |
| TCGA-VS-A94Y | 51 | 144 | 1 | 47 | white | GX | IIB | T2b | MX | NX |
| TCGA-VS-A94Z | 731 | 742 | 0 | 38 | white | G2 | IIB | T2b | MX | NX |
| TCGA-VS-A950 | 505 | 1210 | 0 | 42 | not reported | G3 | IIIA | T3a | MX | NX |
| TCGA-VS-A952 | 837 | 1547 | 0 | 66 | white | G2 | IB1 | T1b | MX | N0 |
| TCGA-VS-A953 | 399 | 477 | 1 | 63 | not reported | GX | IVA | T4 | MX | NX |
| TCGA-VS-A954 | 427 | 1540 | 0 | 67 | not reported | G2 | IIIB | T3b | MX | NX |
| TCGA-VS-A957 | 497 | 1462 | 0 | 64 | white | G3 | IB1 | T1b1 | MX | NX |
| TCGA-VS-A958 | 799 | 1525 | 0 | 46 | white | G2 | IIB | T2b | MX | NX |
| TCGA-VS-A959 | 566 | 1379 | 0 | 76 | not reported | G3 | IIB | T2b | MX | NX |
| TCGA-VS-A9U5 | 536 | 1367 | 0 | 57 | white | G3 | IIB | T2b | MX | NX |
| TCGA-VS-A9U6 | 895 | 1320 | 0 | 52 | white | GX | IVB | T4 | M1 | NX |
| TCGA-VS-A9U7 | 819 | 1290 | 0 | 30 | white | G3 | IB1 | T1b | M0 | N1 |
| TCGA-VS-A9UB | 282 | 648 | 0 | 54 | not reported | G3 | IIB | T2b | MX | N0 |
| TCGA-VS-A9UC | 690 | 691 | 0 | 32 | not reported | G2 | IIB | T2b | MX | NX |
| TCGA-VS-A9UD | 401 | 599 | 0 | 73 | white | G2 | IIIA | T3a | MX | NX |
| TCGA-VS-A9UH | 260 | 1427 | 0 | 53 | black or african american | GX | IVA | T4 | MX | NX |
| TCGA-VS-A9UI | 867 | 1246 | 0 | 76 | white | GX | IIB | T2b | MX | NX |
| TCGA-VS-A9UJ | 335 | 52 | 1 | 55 | not reported | GX | IIB | T2b | MX | NX |
| TCGA-VS-A9UL | 31 | 442 | 1 | 79 | white | G3 | IIIB | T3b | MX | NX |
| TCGA-VS-A9UM | 749 | 829 | 1 | 39 | black or african american | G2 | IVB | T3b | MX | NX |
| TCGA-VS-A9UO | 865 | 1260 | 0 | 43 | white | G2 | IIB | T2b | MX | N0 |
| TCGA-VS-A9UP | 851 | 1316 | 0 | 43 | not reported | G3 | IIA | T2a | MX | NX |
| TCGA-VS-A9UQ | 449 | 1094 | 0 | 32 | not reported | G2 | IB | T1b | MX | N0 |
| TCGA-VS-A9UR | 805 | 611 | 0 | 53 | white | GX | IIA | T2a | MX | NX |
| TCGA-VS-A9UT | 886 | 482 | 0 | 72 | asian | G3 | IB | T1b1 | MX | N1 |
| TCGA-VS-A9UU | 70 | 442 | 0 | 42 | not reported | G1 | IIB | T2b | MX | NX |
| TCGA-VS-A9UV | 260 | 104 | 1 | 74 | not reported | GX | IVA | T4 | MX | NX |
| TCGA-VS-A9UY | 618 | 555 | 1 | 29 | not reported | G2 | IVB | T1b2 | MX | NX |
| TCGA-VS-A9UZ | 525 | 2044 | 0 | 61 | white | G2 | IB1 | T1b | MX | N0 |
| TCGA-VS-A9V0 | 257 | 573 | 0 | 58 | not reported | G3 | IB | T1b | MX | NX |
| TCGA-VS-A9V1 | 151 | 157 | 1 | 46 | white | G2 | IVB | T3b | MX | NX |
| TCGA-VS-A9V2 | 702 | 427 | 0 | 29 | white | G2 | IB1 | T1b | MX | N0 |
| TCGA-VS-A9V3 | 519 | 453 | 0 | 62 | white | G3 | IVB | T4 | MX | NX |
| TCGA-VS-A9V4 | 626 | 132 | 1 | 63 | white | G2 | IVA | T4 | MX | NX |
| TCGA-VS-A9V5 | 533 | 494 | 1 | 50 | white | G2 | IIB | T2b | MX | NX |
| TCGA-VS-AA62 | 296 | 469 | 1 | 51 | white | G2 | IIB | T2b | MX | NX |
| TCGA-WL-A834 | 363 | 791 | 0 | 57 | white | G3 | unknow | T1b1 | unknow | N1 |
| TCGA-XS-A8TJ | 909 | 890 | 0 | 41 | black or african american | G2 | IB1 | T1b1 | M0 | N1 |
| TCGA-ZJ-A8QO | 921 | 0 | 0 | 73 | not reported | unknow | unknow | unknow | unknow | unknow |
| TCGA-ZJ-A8QQ | 540 | 2056 | 0 | 24 | white | GX | IIB | T1b2 | M0 | N0 |
| TCGA-ZJ-A8QR | 834 | 582 | 1 | 38 | native hawaiian | GX | I | TX | MX | NX |
| TCGA-ZJ-AAX4 | 518 | 21 | 0 | 85 | white | G3 | II | T2 | M0 | N0 |
| TCGA-ZJ-AAX8 | 905 | 0 | 0 | 58 | not reported | G2 | IIIB | T3b | M0 | N1 |
| TCGA-ZJ-AAXA | 322 | 43 | 0 | 64 | white | G2 | IB1 | T1b | MX | N0 |
| TCGA-ZJ-AAXB | 870 | 0 | 0 | 42 | white | G3 | IB2 | T1b2 | M0 | N0 |
| TCGA-ZJ-AAXD | 979 | 0 | 0 | 35 | not reported | G2 | IIIB | T3b | M0 | N0 |
| TCGA-ZJ-AAXF | 801 | 0 | 0 | 62 | not reported | G3 | IIB | T2b | M0 | N0 |
| TCGA-ZJ-AAXI | 180 | 0 | 0 | 67 | not reported | G2 | IIB | T2b | M0 | NX |
| TCGA-ZJ-AAXJ | 515 | 0 | 0 | 43 | not reported | unknow | IIB | T2b | MX | N0 |
| TCGA-ZJ-AAXN | 394 | 0 | 0 | 34 | white | unknow | IB2 | T1b2 | M0 | N1 |
| TCGA-ZJ-AAXT | 698 | 0 | 0 | 54 | white | G2 | IIIB | T3b | M0 | N1 |
| TCGA-ZJ-AAXU | 15 | 5 | 0 | 51 | white | G2 | IIB | T2b | M0 | N0 |
| TCGA-ZJ-AB0H | 869 | 0 | 0 | 48 | not reported | unknow | IIIB | T3b | M0 | N1 |
| TCGA-ZJ-AB0I | 978 | 0 | 0 | 25 | white | unknow | IIB | T2b | MX | N1 |
| TCGA-ZX-AA5X | 259 | 119 | 0 | 64 | white | G2 | IIIB | TX | MX | NX |
